# Exploring the hypothetical role of *Bacteroides* species in depression progression: insights from metagenomic analysis

**DOI:** 10.1128/spectrum.03153-24

**Published:** 2026-08-11

**Authors:** Zihua Li, Jingxi Sun, Jinlong Yang, Panpan Han, Liping Min, Yongkang Cheng, Yuanqiang Zou, Zhuhua Liu

**Affiliations:** 1 School of Basic Medical Sciences, Ningxia Medical University 105002, Yinchuan, China; 2 College of Life Sciences, University of Chinese Academy of Sciences 74519, Beijing, China; 3 College of Forensic Science, Xi’an Jiaotong University, Xi’an, Shaanxi, China; 4 Mental Health Center, General Hospital of Ningxia Medical University 74747, Yinchuan, Ningxia, China; 5 Laboratory of Genomics and Molecular Biomedicine, Department of Biology, University of Copenhagen 4321, Copenhagen, Denmark; The Chinese University of Hong Kong, Hong Kong, Hong Kong; Chinese Academy of Sciences, Beijing, China; Yerevan State University, Yerevan, Armenia

**Keywords:** depression, metagenomics, fecal microbiome, *Bacteroides*, Random Forest

## Abstract

Depression, a psychiatric disorder with significant morbidity and mortality, has a complex etiology. Recent advances in microbiome research have highlighted the potential role of fecal microbiota in depression pathogenesis. This study utilized shotgun metagenomic sequencing to compare the fecal microbiota of 28 depression patients and 26 healthy individuals. Significant differences in fecal microbiota composition were observed between the two groups. We generated 350 non-redundant high-quality metagenome-assembled genomes (MAGs) by binning and conducted comparisons between the depression and control groups. Notably, we found that the MAGs enriched in people with depression mostly belonged to *Bacteroides,* indicating a close link between *Bacteroides* abundance and the development of depression, suggesting that *Bacteroides* might be a potential culprit for depression. In the depression group, we found that the module of nitric oxide synthesis was remarkably enriched, and all *Bacteroides* MAGs contained genes annotated as nitric oxide synthase, suggesting that increased levels of *Bacteroides* may contribute to elevated nitric oxide synthesis. A distinct microbial signature consisting of *Arthrobacter* sp.*_U41*, *Bacillus cereus*, *Campylobacter rectus,* and *Pasteurella dagmatis* accurately discriminates between depressed individuals and healthy controls, achieving an average area under the receiver operating characteristic curve of 0.950. This research sheds light on the potential role of fecal microbiota in depression and highlights specific metabolic pathways and microbial markers for further investigation.

**IMPORTANCE:**

This research highlighted significant differences in the composition and function of fecal microbiota between individuals with depression and healthy individuals, particularly the enrichment of *Bacteroides* metagenome-assembled genomes (MAGs) in depression patients. The upregulation of the nitric oxide synthesis pathway associated with these MAGs belonging to *Bacteroides* in the gut of depression patients had also been observed. The selected bacterial biomarkers reliably differentiate depression cases from healthy controls with high diagnostic accuracy (mean area under the receiver operating characteristic curve = 0.950). Our results suggest the importance of exploring microbial markers as potential diagnostic and therapeutic targets in managing depression.

## INTRODUCTION

Depression, as a prevalent neuropsychiatric disease, is not only associated with high treatment costs but also characterized by high incidence and mortality rates. It often undermines individuals’ psychological resilience and significantly increases the risk of suicide (1). Globally, depression has a wide range of impacts, affecting approximately 322 million people, or 4.4% of the global population, with a prevalence rate exceeding the natural growth rate of the global population (2). All Bacteroides metagenome-assembled genomes (MAGs) were annotated as nitric oxide synthase. This disease is soaring globally, and its extensive influence has made it an important issue in the field of public health. According to relevant statistics, the lifetime prevalence of depression is as high as 15%, making it a major culprit endangering human health (3).

The pathogenesis of depression is intricate and has yet to be fully elucidated, intricately intertwined with complex psychological and physiological reactions. Recently, Vinelli et al. (4) emphasized the pivotal role of the human fecal microbiota in maintaining health, uncovering the extensive functionality of these tiny organisms, their similarity in function to host organs, and the depth of their interactions. Notably, the intimate connection between fecal microbiota and the brain via the microbiota-gut-brain axis offers a novel perspective for understanding the pathophysiology of mental disorders, including depression (5). Compared to healthy individuals, the fecal microbiota composition in patients with depression is markedly distinct, characterized by an increased ratio of Bacteroidetes to Firmicutes, as evidenced by studies conducted by Zheng et al. (6) and Simpson et al. (7), underscoring the potential role of fecal microbiota in depression pathology. By intricately modulating endocrine, immune, and neuroactive pathways, the microbiota profoundly influences the communication mechanisms between the gut and the brain. This process involves (i) the hypothalamic-pituitary-adrenal (HPA) axis, where microbial metabolites regulate cortisol secretion and stress responses (8); (ii) immune modulation through cytokine signaling (e.g., IL-6 and TNF-α) that affects blood-brain barrier permeability and neuroinflammation (9); (iii) neurotransmitter systems such as γ-aminobutyric acid, which are directly synthesized by gut bacteria or indirectly regulated through microbial influence on host synthesis (10); (iv) microbial metabolites, including short-chain fatty acids (SCFAs) and bile acids, that alter brain-derived neurotrophic factor (BDNF) levels (11).

In the investigation of the relationship between depression and fecal microbiota, traditional 16S rDNA sequencing has been widely applied. However, its limitation lies in identifying microbial taxa only up to the genus level, thus unable to precisely capture interspecies differences and functional or metabolic characteristics. To address this limitation, the introduction of metagenomics becomes paramount. Compared to 16S rDNA sequencing, metagenomics provides a robust tool for comprehensively deciphering the complex interactions between hosts and microbiota. Metagenomic assembly enables the reconstruction of draft genomes of bacteria, archaea, and viruses that are uncultivable in laboratory settings, facilitating strain-level gene and functional annotation, comparative genomic analysis, and evolutionary studies. This approach provides insights into the ecological adaptation mechanisms, nutritional interactions, metabolic functions, and other traits of these uncultivable strains. Moreover, it facilitates the investigation of key microbial species that play crucial roles in complex diseases, as well as the interaction mechanisms between pathogens and hosts, including their microevolutionary processes. At present, fecal microbiota in depression remains scarce (6, 7, 12). Building upon this backdrop, the present study employs metagenomic approaches to systematically dissect the fecal microbiota structure of healthy individuals and patients with depression. It not only reveals significant differences in fecal microbiota composition between the two groups but also identifies specific bacteria or microbial community structures associated with depression characteristics, thereby opening new avenues for a deeper understanding of the pathogenesis of depression.

In this study, we highlighted significant differences in the composition and function of fecal microbiota between patients with depression and healthy individuals, particularly the enrichment of *Bacteroides* MAGs in depressed patients. We also observed an upregulation of the nitric oxide (NO) synthesis pathway associated with these *Bacteroides* MAGs in the gut of depressed patients. The identification of a specific set of bacteria (average area under the receiver operating characteristic [ROC] curve [AUC] value = 0.950) accurately distinguishes individuals with depression from healthy individuals. Our findings underscore the importance of exploring microbial markers as potential diagnostic and therapeutic targets for treating depression.

## MATERIALS AND METHODS

### Participant selection

#### Subject recruitment

The fecal samples were collected at the General Hospital of Ningxia Medical University. The study was conducted from January 2022 to June 2023, during which patients with depression at the Mental Health Center of Ningxia Medical University General Hospital were included in the study. The healthy control group was composed of senior students and graduate students from Ningxia Medical University. In this study, a total of 54 participants were recruited, of whom 28 were diagnosed with depression, and 26 were healthy controls. The participants were aged between 18 and 49 years old, with a male-to-female ratio of 27:27. The patients’ depression was diagnosed according to the strict criteria of the Self-Rating Depression Scale (SDS), where a score of more than 53 indicates the presence of such a condition. The fluctuation range of SDS scores for patients with depression is 53.75–112.5, and the average SDS score is 87.32. Fecal samples were collected from all participants immediately and stored at −80°C.

#### Inclusion criteria (case group)

Participants were aged 18–60 years and were outpatients with a first onset of the disease who had either not taken any psychotropic drugs or had stopped taking them for 2 weeks or longer before enrollment. Participants were required to meet the diagnostic criteria for the corresponding diseases defined in the Diagnostic and Statistical Manual of Mental Disorders, Fifth Edition, and the International Statistical Classification of Diseases and Related Health Problems, Implementation (ICD-11).

#### Healthy control group

Participants had no record of any mental disorder or major negative life events and no history of alcohol or drug abuse.

#### Exclusion criteria

Subjects were excluded if they were under 18 years old or over 60 years old; had secondary mental disorders, such as those induced by certain medications or severe physical illnesses; had a history of alcoholism or drug abuse; had organic lesions or neurodegenerative diseases of the central nervous system, including stroke, Alzheimer’s disease, or Parkinson’s disease; had accompanying severe physical illnesses; and had a history of head injury, brain parenchymal disease, or brain tumors. The female subjects were excluded if they were menstruating, pregnant, or lactating.

### Stool metagenomic sequencing and metagenomic profile

We conducted shotgun metagenomic sequencing on fecal samples collected from 54 participants to obtain a comprehensive metagenomic profile. Initially, DNA extraction from the fecal samples was performed using the MagPure Stool DNA KF Kit55. Subsequently, shotgun sequencing was carried out on the DNBSEQ-T7 platform with 100 bp paired-end reads. Following this, raw data from all samples underwent quality control using fastp, which involved trimming poly-G and poly-X sequences, cutting front and tail sequences, and applying a low complexity filter. Human-derived sequences were then removed using Bowtie2 to generate clean data. The average sequencing depth achieved was 115,606,258.7. Subsequently, the clean data were processed with Kraken2 to determine the microbial composition at the phylum, genus, and species levels, utilizing default parameters for analysis.

### Fecal microbiome functional analysis

The functional analysis of the fecal microbiome was conducted using a gene set construction approach, comprising the following main steps: (i) assembly of clean data into contigs using Megahit, with parameters for paired-end reads and multi-threading; (ii) gene prediction on the assembled contigs for each sample using Prodigal, applying parameters suitable (-p meta) for metagenomic data; (iii) merging predicted gene sequences from all samples and removing redundant sequences with CD-HIT, resulting in a non-redundant gene set containing 1,729,639 sequences; (iv) functional annotation of all sequences in the gene set after conversion to protein sequences using EggNOG-mapper, employing parameters for efficient annotation. This process yielded distinct profiles for both genes and metabolic pathways. A chi-square test (corrected by false discovery rate [FDR]) was then performed to identify differentially expressed genes identified between patients with depression and healthy individuals. In addition, linear discriminant analysis (LDA) was then performed to identify differentially enriched metabolic pathways between patients with depression and healthy individuals (LDA >2). Finally, Omixer-RPM was utilized to predict gut-brain modules for the differential genes in the two gut microbiomes.

### Binning metagenomic data, abundance calculation of MAGs, and functional annotation of MAGs

We performed metagenomic binning using three complementary tools: MetaBAT2, MaxBin2, and CONCOCT. Initial binning generated 12,858 MAGs. Quality assessment was conducted using CheckM2, retaining 9,115 medium-to-high quality MAGs (completeness >70% and contamination <10%). Redundant MAGs were removed with dRep (similarity threshold, 99%), yielding 3,759 non-redundant MAGs. Subsequent clustering at 95% average nucleotide identity using dRep further refined the data set to 350 high-quality representative MAGs. Taxonomic classification was performed with GTDB-Tk (classify_wf module) based on the Genome Taxonomy Database (GTDB, r214). Phylogenetic tree construction utilized the GTDB-Tk-inferred taxonomy and was visualized via the Interactive Tree of Life platform (https://itol.embl.de). Finally, a custom Kraken2 database was built using the kraken2-build module to quantify MAG abundances across samples.

### Random Forest-based feature selection and model evaluation

#### Feature importance ranking

To identify the most predictive features, we employed a Random Forest (RF) algorithm to compute feature importance scores. Features were ranked based on their contribution to model prediction, allowing us to eliminate irrelevant or redundant variables, thereby reducing model complexity and mitigating overfitting risks.

#### K-fold cross-validation for optimal feature subset

We further optimized feature selection using k-fold cross-validation to determine the best-performing feature subset: the data set was partitioned into five equally sized folds. In each iteration, four folds were used for training, and the remaining one fold served as the validation set. The mean cross-validation error across all iterations was computed for different feature subsets. The feature subset yielding the lowest mean error was selected as the optimal combination.

#### Model performance assessment for Random Forest (ROC-AUC)

To evaluate the Random Forest model’s generalization ability, the data set was randomly split into a 70% training set and a 30% testing set. The RF model was trained on the optimal feature subset, and its performance was assessed using the area under the ROC curve. To ensure robustness, this entire process (data splitting, model training, and performance evaluation) was repeated 1,000 times with different random partitions. This repeated random subsampling validation strategy was adapted from the framework described by Petit III et al. (13) for evaluating classifier performance under varying data partitions. The mean AUC across all iterations was reported as the final performance metric for the RF model.

#### Validation using alternative algorithms

To verify the predictive capability and interpretability of the combined marker derived from the RF model’s optimal feature subset, we introduced Support Vector Machine (SVM) and Logistic Regression (LR) algorithms. Employing the same rigorous assessment framework, the data set was similarly randomly divided (70% training and 30% test) for each alternative algorithm. The SVM and LR models were then trained on the same optimal feature subset identified for the RF model. Their performance was likewise evaluated using AUC. Consistent with the RF evaluation, this process for each alternative model (SVM and LR) was repeated 1,000 times with different random splits, and the mean AUC was reported as their respective final performance metric.

### Statistical analysis

The sequencing data underwent a Wilcoxon test for group differences, while other data were analyzed using analysis of variance or chi-square tests, and we applied multiple testing correction (FDR adjustment) to avoid false positives, with statistical significance set at adj*. P* < 0.05. Random Forest prediction was executed using the randomForest package in R (v 3.1.1). Additionally, β diversity and β diversity analyses were conducted using the vegan package in R (v 3.1.1). We used corrplot to calculate the correlation coefficient and generate the heatmap.

It is noteworthy that, due to the absence of significant differences in fecal microbiota composition between individuals with mild depression and healthy controls, we sought to minimize confounding factors by selecting 25 patients with depression and 26 healthy microbiota samples for subsequent functional analyses of the fecal microbiome and Random Forest modeling.

## RESULTS

### Characteristics of study population

At the General Hospital of Ningxia Medical University, we rigorously collected fecal samples from three patients with mild depression, as indicated by a Self-Rating Depression Scale score exceeding 53, and from 25 patients with major depressive disorder, with scores exceeding 73. Simultaneously, we collected fecal samples from 26 healthy volunteers, and the detailed SDS scores of the collected depression patients could be found in Table 1. Following shotgun sequencing of these fecal samples, we obtained a total data volume of 304GB, with the largest sample data size being 9.2GB, and the smallest size being 3.8GB. The average data volume per sample was approximately 5.67 ± 0.15GB. After removing human-derived genic data from the metagenomic data, the data volume was reduced to 293GB, with the largest sample data size at 8G and the smallest at 3.7GB. The average data volume per sample was approximately 5.46 ± 0.14GB.

**TABLE 1:** Detailed SDS scores of collected depression patients^*a*^^,^^*b*^

Collection number	Sequencing number	SDS score	Severity level
WXY-P21	MDD1	112.5	Major
LJY-P24	MDD2	106.25	Major
TXR-P19	MDD3	102.5	Major
CYC-P12	MDD4	100	Major
HP-P31	MDD5	100	Major
MRM-P3	MDD6	100	Major
JCH-P11	MDD7	96.25	Major
BQ-P26	MDD8	95	Major
MK-P13	MDD9	95	Major
ZLH-P17	MDD10	95	Major
ZSX-P25	MDD11	95	Major
ZXM-P22	MDD12	93.75	Major
ZYZ-P29	MDD13	93.75	Major
ZY-P27	MDD14	91.25	Major
LML-P9	MDD15	88.75	Major
MQ-P16	MDD16	87.5	Major
GF-P4	MDD17	85	Major
WQH-P10	MDD18	83.75	Major
JFR-P15	MDD19	82.5	Major
LHC-P1	MDD20	82.5	Major
LX-P30	MDD21	78.75	Major
WXZ-P14	MDD22	78.75	Major
WYM-P20	MDD23	78.75	Major
HHK-P6	MDD24	77.5	Major
XJB-P28	MDD25	76.25	Major
MWM-P23	MD3	58.75	Mild
MRP-P8	MD1	56.25	Mild
JYP-P7	MD2	53.75	Mild

^*a*^ The table provides detailed information on the Self-Rating Depression Scale scores of the collected depression patients. The table includes sample ID, sequencing ID, severity level of depression, and the corresponding SDS score for each patient. The sequencing ID uniquely identifies each patient’s fecal sample. The severity level is categorized as either “Mild” or “Major” based on the SDS score.

^*b*^ MDD, major depressive disorder; MD, mild depression; and HC, healthy control.

### Fecal microbiota disparities between depression patients and healthy controls

**Fig 1:**
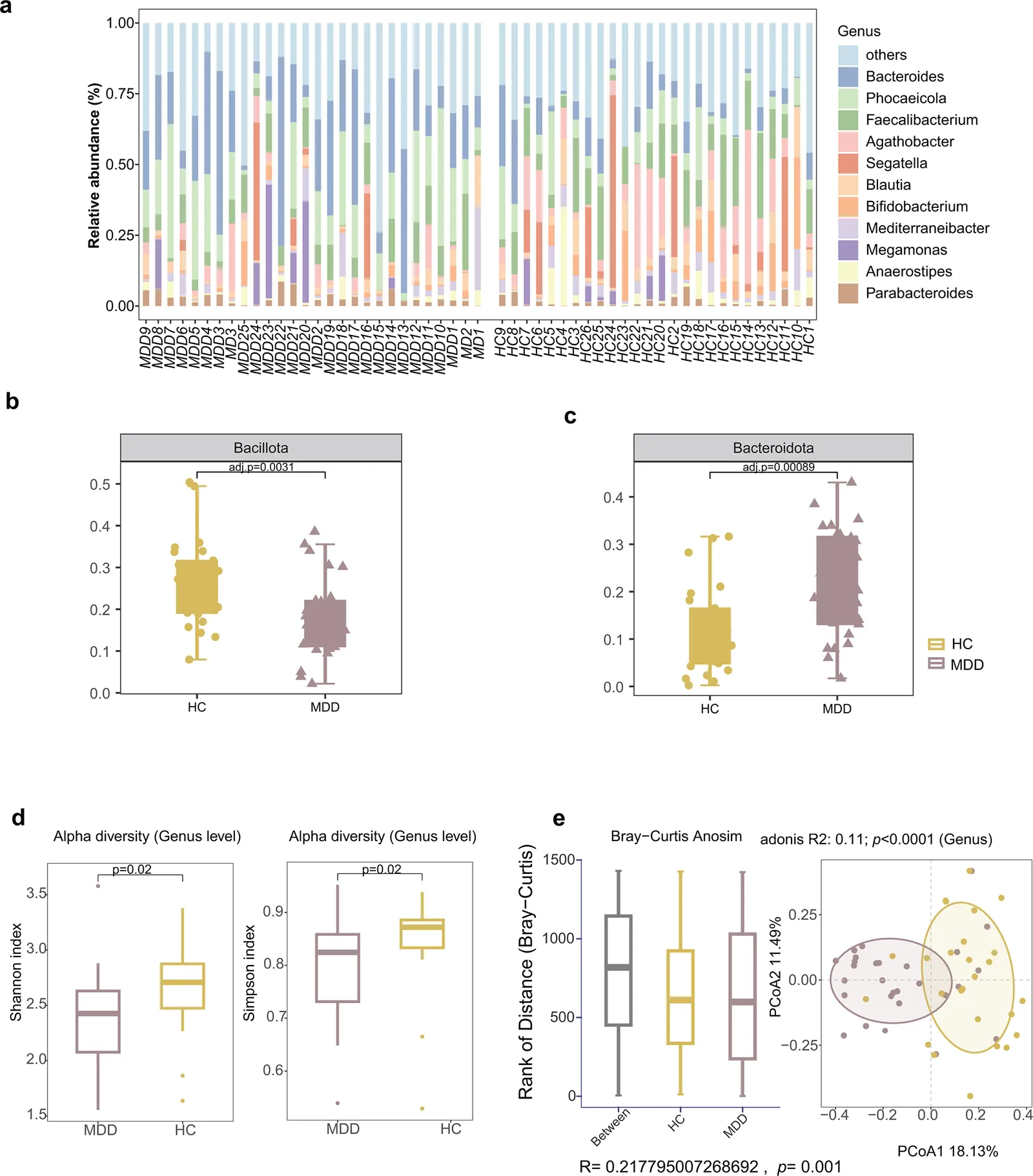
Fecal microbiota composition analysis in depression people and healthy controls. (**a**) Stacked bar chart illustrating the taxonomic composition at the genus level for each sample. (**b and c**) Genera significantly enriched in the fecal microbiota between depression patients and healthy controls (Wilcoxon test, adj.*P* < 0.05). (**d**) The difference in Shannon index and Simpson index at the genus level in the depression and control groups (Wilcoxon test, adj.*P* < 0.05). (**e**) The difference in β diversity by principal coordinate analysis at the genus level in the depression and control groups (PERMANOVA, *P* < 0.05). Yellow represents the healthy group, while purple represents the disease group. *adj.*P* < 0.05; **adj.*P* < 0.001.

The gut metagenomics data of 28 depression patients and 26 healthy individuals were analyzed to investigate potential differences in fecal microbiota composition and structure between the two cohorts. We identified *Bacteroides* (15.83%)*, Phocaeicola* (12.77%), *Faecalibacterium* (10.65%)*, Agathobacter* (7.78%)*, Segatella* (5.94%), and *Blautia* (4.55%) as the most prevalent taxa at the genus level in all individuals; however, there were big differences in the relative abundance of dominant bacterial genera among the samples (Fig. 1a). Subsequent Wilcoxon rank-sum tests at the phylum level demonstrated a significant rise in the relative abundance of *Bacteroidota* and a marked decline in *Bacillota* in the fecal microbiota of depression patients in comparison to healthy controls (Fig. 1b, adj.*P =* 0.0031, *W* = 530; Fig. 1c, adj*.P* = 0.00089, *W* = 160; Table S1). We used the Shannon and Simpson indices to assess the α-diversity of the fecal microbiota at the genus level and conducted principal coordinates analysis to reduce dimensionality and cluster the fecal microbiota data, aiming to evaluate the structure of the fecal microbiota at the genus level. The results showed significantly lower Shannon and Simpson indices in the fecal microbiota of depression patients compared to healthy controls (Fig. 1d [right], *P* = 0.01298, *W* = 220; Fig. 1d [left], *P* = 0.01236, *W* = 219). Additionally, the structure of the fecal microbiota at the genus level exhibited significant differences between depression patients and healthy individuals [Fig. 1e, PERMANOVA: *F*(1,52) = 3.99, *P* < 0.0001]. These results emphasized clear compositional disparities in the fecal microbiota between depression patients and healthy controls. An intriguing observation from our study was the absence of significant differences in the fecal microbiota composition between individuals with mild depression and healthy controls (Fig. S1). To mitigate potential interference in subsequent statistical analyses, samples from individuals with mild depression were excluded from further study.

### Differential functional profiles of fecal microbiota between depression patients and healthy individuals

**Fig 2:**
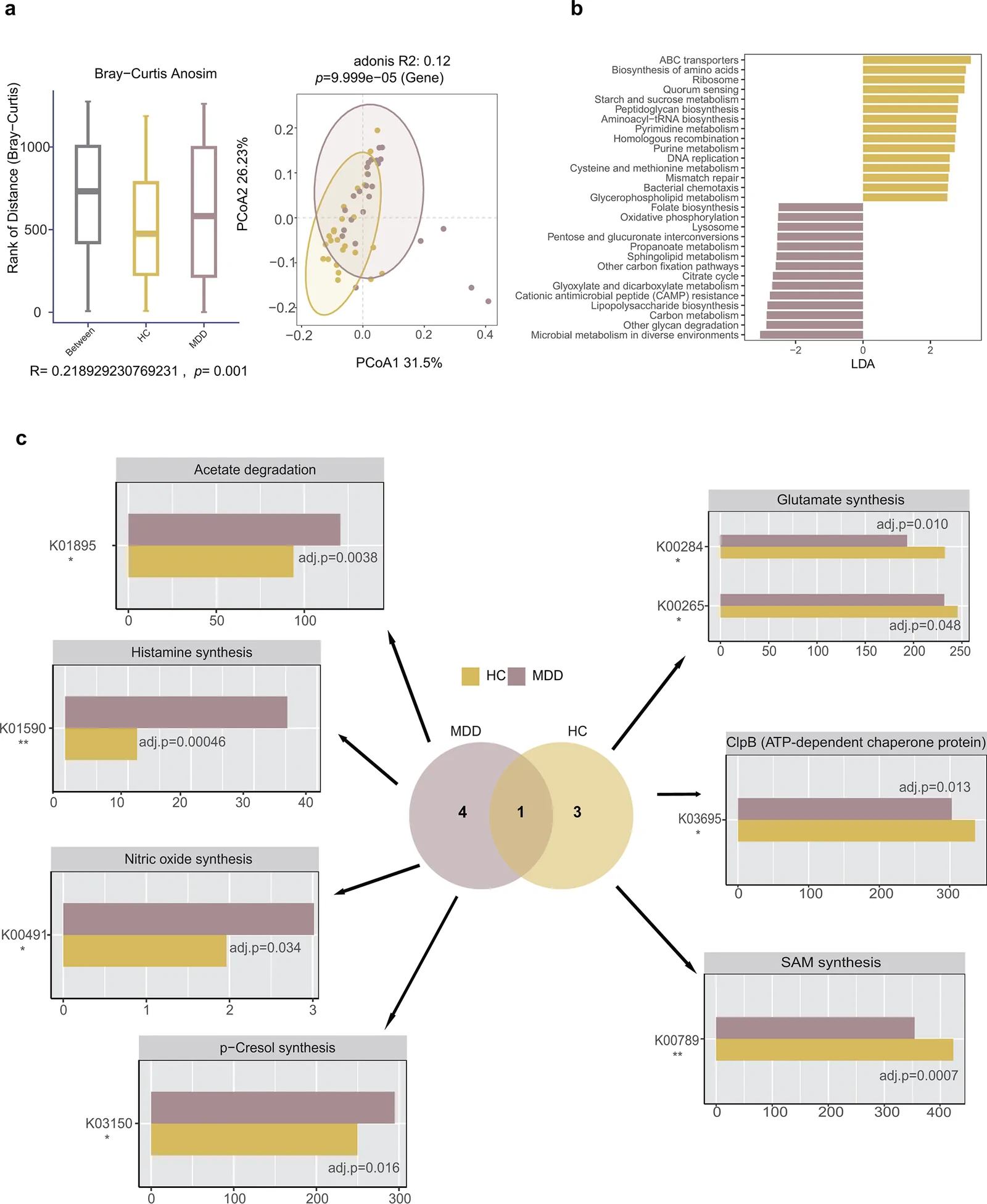
Fecal microbiota functional analysis in depression patients and healthy people. (**a**) The difference in β diversity by principal coordinate analysis at the gene level in the depression and control groups (PERMANOVA, *P* < 0.05). (**b**) Differentially enriched metabolic pathways in fecal microbiota of depression patients vs healthy controls. (**c**) Metabolic module maps of the fecal microbiota in both groups. The bar graph title indicates the gene’s gut-brain module and displays only genes with significant differences in the depression and control groups (chi-square test, adj.*P* < 0.05). Yellow represents the healthy group, while purple represents the disease group. *adj.*P* < 0.05; **adj.*P* < 0.001.

In our effort to elucidate the functional differences of the fecal microbiota in individuals with depression, we conducted a comprehensive exploration of microbial functions by building functional gene sets. Our methodology involved the genome assembly, gene prediction, and functional annotation of genes, culminating in the creation of self-building and non-redundant functional gene sets. Subsequently, we quantified the abundance of all functional genes in each sample and conducted principal coordinates analysis at the gene level to compare the two cohorts, unveiling significant differences in the functional profiles of the fecal microbiota between individuals with depression and healthy controls [Fig. 2a, PERMANOVA: *F*(1,49) = 6.95, *P* < 0.0001, Table S2]. Following this, we identified the specific genes exhibiting differential abundance between the two cohorts. Through chi-square tests, we identified 801 genes with remarkably increased abundance in the fecal microbiota of individuals with depression, while 723 genes showed significantly increased abundance in the fecal microbiota of healthy individuals. Next, to identify pathways exhibiting significant differential abundance between healthy individuals and depression patients, we employed non-parametric statistical tests followed by linear discriminant analysis to quantify the effect sizes of these pathways, thereby reflecting the magnitude of their differences, with all analyses performed using the LEfSe software package (https://github.com/SegataLab/lefse). The resulting LDA scores and associated pathways are presented in Fig. 2b.

To further understand the functional modules of these differentially expressed genes, we utilized the omixer-rmp tool to predict metabolic module profiles of the fecal microbiota in both groups. As depicted in Fig. 2c and Table S3, the distinct gene profiles of the fecal microbiota in individuals with depression revealed specific metabolic modules, including acetate degradation, histamine synthesis, nitric oxide synthesis, and p-Cresol synthesis. Conversely, healthy individuals exhibited a different array of metabolic modules associated with glutamate synthesis and ClpB (ATP-dependent chaperone protein) expression. Based on the aforementioned findings, it could be concluded that there were significant differences in the fecal microbiota between healthy individuals and patients with depression. The comparison of gene abundance levels and the prediction of metabolic modules revealed distinct functional characteristics between the fecal microbiota of the two groups.

### Characterization of enriched MAGs in fecal microbiota of healthy individuals and patients with depression

**Fig 3:**
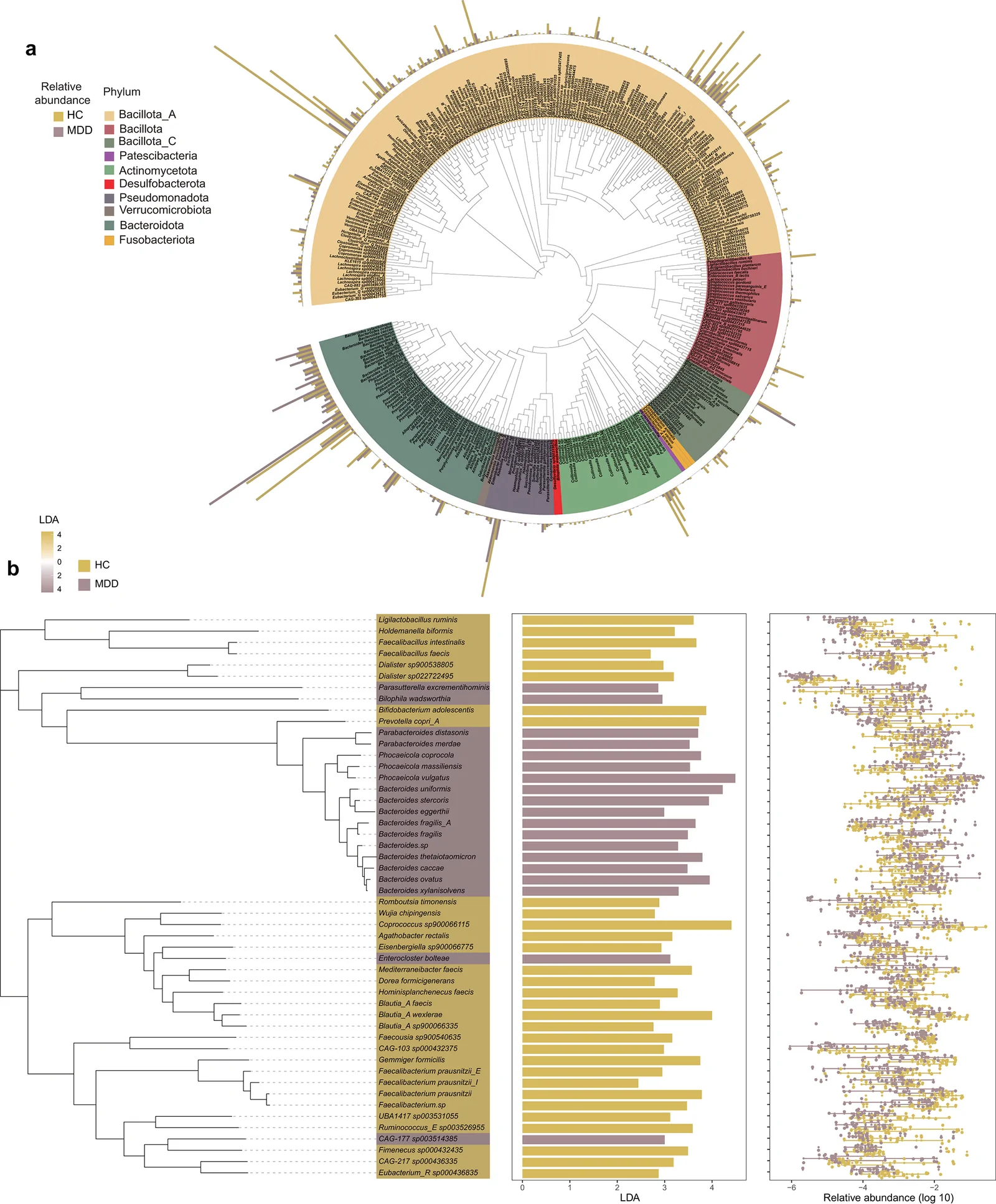
Comparative analysis of MAGs enriched in the fecal microbiota of depression patients and healthy individuals. (**a**) Evolutionary tree of MAGs, with different colors representing different phyla. (Left) (**b**) Evolutionary tree of MAGs significantly enriched in the depression and control groups, with yellow indicating MAGs enriched in the gut of depression patients and purple representing MAGs enriched in the gut of healthy individuals. (Right) (**b**) The bar graph represents the LDA values of two significantly different groups of MAGs, while the boxplot represents the relative abundance of MAGs.

**Fig 4:**
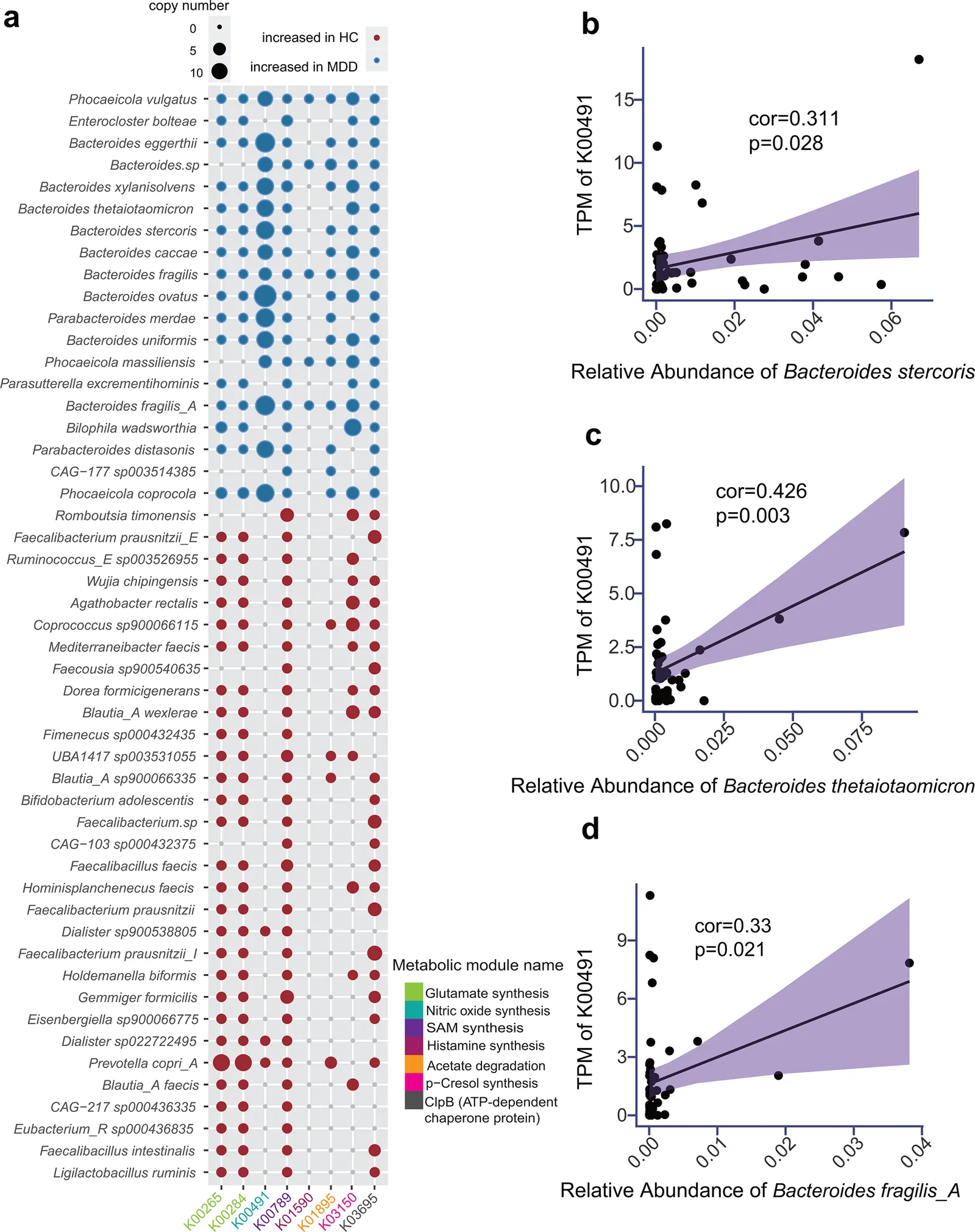
Gene prediction, functional annotation with enriched MAGs, and linear correlation analysis with gene K00491 abundance in Bacteroides species. (**a**) Gene prediction and functional annotation on MAGs significantly enriched in the depression group and control group, with this figure only displaying genes consistent with those in Fig. 2b; (**b–d**) Linear correlation results among *Bacteroides stercoris*, *Bacteroides thetaiotaomicron*, and *Bacteroides fragilis_A* with abundance of gene K00491.

To characterize depression-associated bacteria, we performed metagenomic assembly and binning using Concoct, MaxBin2, and MetaBAT2, yielding 9,115 MAGs. Quality filtering (≥70% completeness and ≤10% contamination) retained 3,759 medium-/high-quality MAGs, which were dereplicated to 350 non-redundant MAGs using dRep. These MAGs were taxonomically annotated against GTDB (Table S4) and quantified via Kraken2. Their phylogenetic relationships are shown in Fig. 3a. The results showed 31 MAGs were enriched in the fecal microbiota of healthy individuals, whereas 19 MAGs were enriched in the fecal microbiota of patients with depression. The specific taxonomic information of these MAGs and their LDA values are presented in Fig. 3b and Table S5. Interestingly, in the gut of patients with depression, most of the enriched species were taxonomized to the *Bacteroides* species, suggesting that changes in the abundance of species with *Bacteroides* might be associated with the development of depression. To elucidate the specific functions of each MAG enriched in the fecal microbiota of healthy individuals and patients with depression, we conducted gene prediction and functional annotation on these MAGs sequentially. The final results are depicted in Fig. 4a. The gene K00491 (nitric oxide synthase), which is responsible for carbon monoxide production, was found in all MAGs taxonomized into *Bacteroides*. Linear correlation analysis was conducted to examine the linear correlation between the abundance of these *Bacteroides* MAGs and the K00491 gene abundance, as shown in Fig. 4b and c. *Bacteroides stercoris* MAG*, Bacteroides thetaiotaomicron* MAG, and *Bacteroides fragilis* A MAG all exhibited significant positive linear correlations with the abundance of the K00491 gene. This suggests that the increased abundance of *Bacteroides* MAGs in the gut of patients with depression might lead to an elevation in nitric oxide synthesis. This finding aligned with the conclusion in Stool metagenomic sequencing and metagenomic profile, indicating a significantly enhanced capacity of fecal microbiota in patients with depression to synthesize nitric oxide. Moreover, we examined the correlation between SDS scores in patients with depression and these differential MAGs, revealing a significant positive association between the *Bacteroides stercoris* MAG and SDS scores (Fig. S2). The aforementioned research results underscored the dysregulation of the gut microbiota in individuals with depression compared to those who were healthy, and the enrichment of beneficial bacteria in healthy individuals could contribute to the maintenance of gut health and prevent depression, while the significant increase in *Bacteroides* species (*Bacteroides caccae, Bacteroides uniformis, Bacteroides fragilis, Bacteroides xylanisolvens, Bacteroides thetaiotaomicron, Bacteroides eggerthii, Bacteroides ovatus,* and *Bacteroides stercoris*) in patients with depression might be associated with the development of the disease.

### Random Forest identifies discriminative microbial features with high AUC

**Fig 5:**
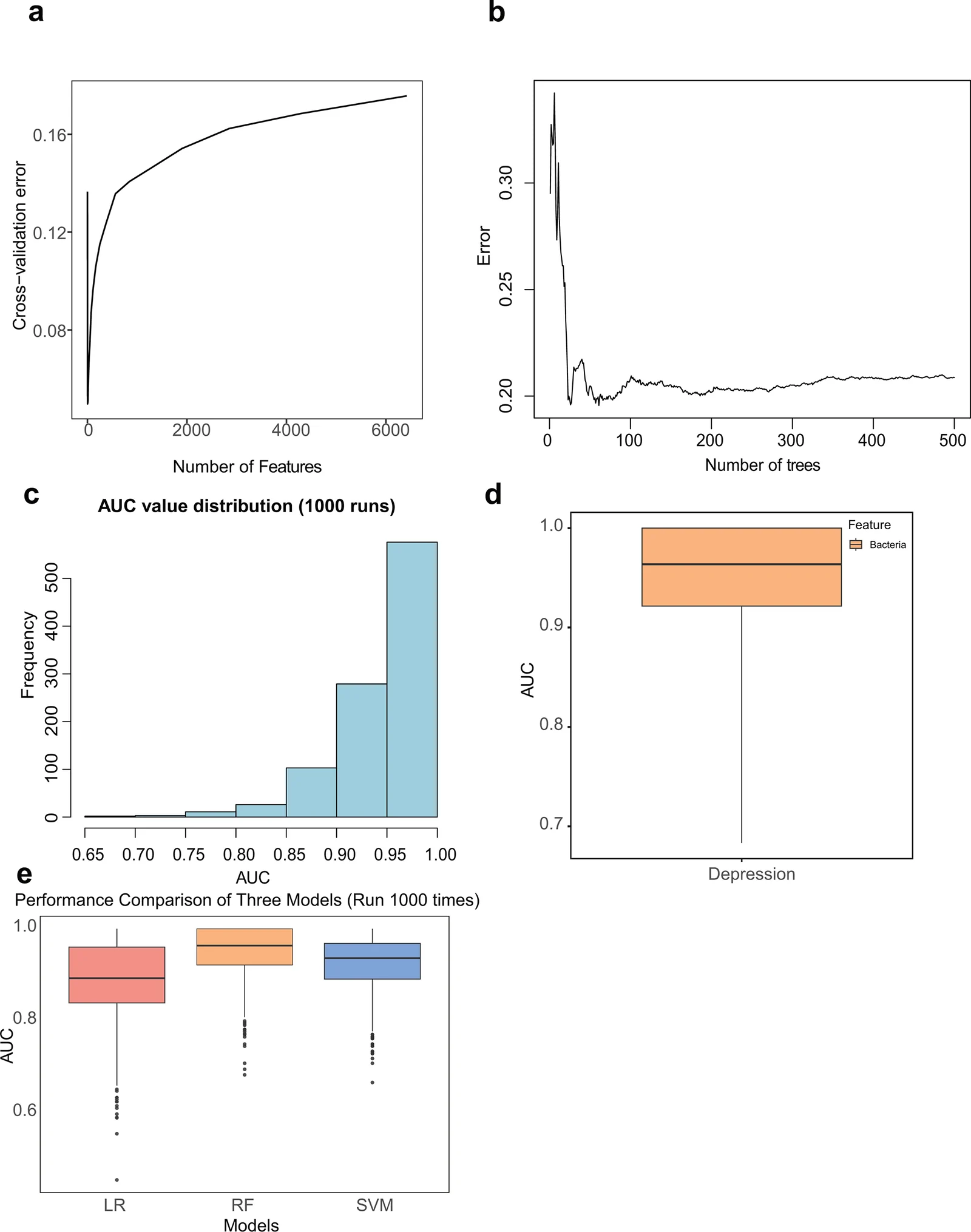
Random Forest model analysis for predicting depressive people. (**a**) Optimal feature subset selection of four microbial taxa via recursive feature selection using Random Forest. (**b**) Effect of the ntree on Random Forest model error rate. (**c**) Distribution of AUC values from 1,000 runs of Random Forest model validation. (**d**) Representative ROC curves of the Random Forest model. (**e**) Comparative AUC performance of Support Vector Machine and Logistic Regression models using the same four-feature subset.

Subsequently, through recursive feature selection with RF, we identified the optimal feature subset consisting of four microbial taxa: *Arthrobacter* sp.*_U41*, *Bacillus cereus*, *Campylobacter rectus,* and *Pasteurella dagmatis*. The model achieved the lowest mean cross-validation error when using these four features, indicating their high discriminative power for the target variable (Fig. 5a, importance scores are shown in Table S6). To optimize the RF model performance, we evaluated the impact of the number of trees (ntree) on prediction stability. The overall error rate stabilized at 500 trees (Fig. 5b), suggesting this as the optimal ensemble size for balancing computational efficiency and predictive accuracy.

Finally, the model was rigorously validated by repeating the training-testing process 1,000 times (70% training and 30% testing). The mean area under the ROC curve (AUC = 0.95) demonstrated excellent classification performance, confirming the robustness of the selected features and the RF model’s generalizability (Fig. 5c and d). Crucially, to assess the intrinsic predictive power and interpretability of this RF-derived feature combination beyond the specific algorithm, we evaluated the same optimal four-feature subset using Support Vector Machine and Logistic Regression models. Employing an identical rigorous validation protocol (repeated 1,000 times with random 70/30 splits), both alternative algorithms achieved high mean AUC values: SVM reached 0.94, and LR achieved 0.92 (Fig. 5e). This consistently high predictive performance across fundamentally different modeling approaches (RF, SVM, and LR) indicates that the discriminative power resides primarily within the selected microbial features themselves, rather than being solely dependent on the RF algorithm. This finding significantly enhances the interpretability and biological relevance of the identified four-taxa biomarker combination.

Furthermore, to substantiate the robustness of our model, we evaluated its discriminative power across distinct subgroups, namely males and females. Our findings reveal that the model performs slightly better within the female subgroup, achieving an average AUC of 0.997 (*N* = 31; 14 healthy controls versus 17 depression patients), whereas the male subgroup attained an average AUC of 0.975 (*N* = 20; 12 healthy controls versus 8 depression patients) (Fig. S3a). These results further affirm the model’s resilience and stability across genders. Additionally, we reincorporated three previously excluded individuals with mild depression into the cohort. The analysis demonstrated that the model maintained a relatively high AUC upon inclusion of mild cases; however, no significant difference was observed compared to the model comprising solely severe depression patients and healthy controls (Fig. S3b). We interpret this observation as partial evidence that these four biomarkers may possess discriminative capacity for mild depression as well. Nonetheless, this conclusion is constrained by the limited sample size of only three mild depression cases.

## DISCUSSION

This study employed shotgun metagenomic sequencing technology and metagenome-assembled genomes to systematically analyze the fecal microbiota of patients with depression and healthy individuals, aiming to explore potential differences in fecal microbiota composition and function between the two groups. The research findings revealed significant discrepancies in gut microbiota composition between patients with depression and normal individuals. Notably, a potential dysbiosis emerged in the fecal microbiota of patients with depression, with changes in the abundance of *Bacteroides*, potentially closely associated with the progression of depression. This study not only contributes to a deeper understanding of the pathogenesis of depression but also has the potential to offer novel biomarkers and targets for the diagnosis and treatment of depression. By elucidating the relationship between fecal microbiota and depression, we can further investigate the role of microbiota in neurological diseases, thereby providing a theoretical basis for developing interventions targeting the gut microbiota.

The pathogenesis of depression was complex, involving changes in both physiological and psychological aspects, and was mainly related to four factors: the HPA axis, the immune system, brain dysfunction, and the gut-brain axis (14, 15). The nearly 100 trillion bacteria in the human intestine affect the development of the immune system. The symbiotic microbiota can affect immune function, nutritional processing, and other physiological aspects of the host (16, 17). Recent studies have revealed the importance of intestinal microbiota in the function of the central nervous system, but the causes and consequences of the changes in intestinal microbiota caused by depression were still unclear (18). Our research findings indicate that there were significant differences in fecal microbiota composition between patients with depression and healthy controls. Notably, the relative abundance of *Bacteroides* significantly increased in the fecal microbiota of depression patients, whereas the relative abundance of *Bacillota* significantly decreased. Furthermore, at the genus level, both the Shannon index and Simpson index of fecal microbiota in depression patients were notably lower than those in healthy controls. Additionally, there existed a marked difference in β-diversity between the fecal microbiota of depression patients and healthy individuals at the genus level. Previous similar reports on major depressive disorder have also found decreased Firmicutes and significantly increased levels of *Bacteroidota*, *Proteobacteria*, and *Actinobacteria* (19). In a previous study, Yang et al. (20) described a significant increase in *Bacteroides* in patients with major depressive disorder. It can be seen that the decrease in the ratio of Firmicutes/Bacteroidetes is a common phenomenon in patients with depression.

Utilizing metagenomic approaches, our analysis revealed significant enrichment of lipopolysaccharide (LPS) biosynthesis in the gut microbiota of depression patients. LPS, a microbial-derived endotoxin extensively documented in previous studies, promotes peripheral inflammation by inducing TNF-α secretion from Schwann cells and rapidly activating microglial cells (21). This cascade ultimately triggers neuroinflammation in the brain, which is related to depression pathogenesis. Then, we further revealed the specific metabolic modules, including acetate degradation, histamine synthesis, nitric oxide synthesis, and p-cresol synthesis of the fecal microbiota in patients with depression. In contrast, the fecal microbiota of healthy individuals exhibited distinct metabolic module arrays associated with glutamate synthesis and the expression of ClpB. This unique metabolic signature pattern observed in depression may be correlated with its pathogenesis. The altered fecal microbiota structure in depression remodels the metabolic patterns of the microbiota, which in turn indirectly influences the metabolic or gene expression patterns of the host. The disturbed fecal microbiota in depression exerts its influence on the gut nervous system through the secretion of metabolites, ultimately affecting brain function and contributing to the development of depression (22). Previously, researchers reported that *Klebsiella* in the gut reduced serum estradiol levels through degradation mediated by 3β-hydroxysteroid dehydrogenase, leading to depressive behavior in mice (23). Similar vertical multi-cohort multi-omics joint studies have revealed that functional changes in fecal microbiota (including various metabolic regulatory pathways such as proline metabolism) were closely related to the occurrence and development of depression (24). It was evident that alterations in fecal microbiota composition led to shifts in microbial metabolic patterns, which were intimately linked to the onset of depression. This observation also suggests that modulating the metabolic patterns of the microbiome may emerge as a novel therapeutic direction for depression.

Our study, employing shotgun metagenomic sequencing and metagenome-assembled genome techniques to explore the gut microbiome, yielded a more profound understanding in contrast to methodologies such as 16S rDNA gene amplicon sequencing or basic read alignment for analysis. This approach not only provides intricate genomic details of bacterial varieties but also enables the correlation of specific microbial genomes with gut-microbial-specific functions. For example, Hibberd et al. (25) pinpointed *Prevotella copri* MAGs as a significant contributor to microbiome-directed complementary food (MDCF-2) induced alterations in metabolic pathways, particularly in the breakdown of polysaccharides found in MDCF-2. Another study delving into the functional aspects of the human gut microbiome revealed several uncultured species with the capacity to break down starch substrates (26). In our research, by comparing the differences in fecal microbiota MAGs between healthy individuals and those with depression, we found that one *Faecalibacterium prausnitzii* MAG was enriched in healthy individuals but significantly depleted in those with depression. *Faecalibacterium*, an anti-inflammatory species, was often downregulated in immune-mediated inflammatory diseases, and these associations may be mediated by the short-chain fatty acid butyrate (27). It has also been reported that *Faecalibacterium*, which produces butyrate, was not observed in patients with depression (28). *Faecalibacterium*, (29) *Ruminococcus*, (30) *Clostridia* (31), and *Lachnospira* (32) were the core microbiome that produces SCFAs in the intestine. Compared with healthy controls, patients with depression syndrome, there is a lack of microbiota that produce SCFAs, which are the products of certain intestinal probiotics and can serve as an energy source for colonic epithelial cells. These acids play crucial roles in the immune system, intestinal barrier, intestinal metabolism, and energy regulation. SCFAs can influence insulin resistance, ischemic stroke, hypercholesterolemia, metabolic diseases, non-alcoholic fatty liver disease (33), rheumatoid arthritis (34), and atopic dermatitis (35–37). Interestingly, our analysis revealed a significant enrichment of the propionate metabolic pathway in the intestines of patients with depression, indicating that propionate, a type of short-chain fatty acid, is more likely to be degraded within this disease cohort. This degradation can further contribute to a decrease in the concentration of short-chain fatty acids in the gut of individuals with depression. It was noteworthy that *Blautia wexlerae* and *Bifidobacterium adolescentis* were significantly depleted in individuals with depression. Studies have shown that oral administration of *Blautia wexlerae* can effectively slow down the progression of diabetes induced by a high-fat diet (38), while *Bifidobacterium adolescentis* is recognized as a producer of γ-aminobutyric acid (39), a neurotransmitter (40) that plays a role in the gut. In the gut of patients with depression, the majority of enriched MAGs belong to the genus *Bacteroides*, suggesting that variations in the abundance of *Bacteroides* species may be linked to the development of depression. Prior studies have reported an increase in the abundance of *Bacteroides* and a decrease in the abundance of Firmicutes and *Lactobacillus* in the fecal microbiota of patients with depression. Stress-induced gut microbiota disturbances lead to reduced release of 5-hydroxytryptamine (serotonin) in the gut, impairing the functions of the prefrontal cortex and hippocampus and resulting in decreased secretion of BDNF and dopamine. These alterations contribute to emotional and cognitive impairments, ultimately triggering depression (41). Our results indicate that the increased abundance of *Bacteroides* MAGs in the gut of patients with depression might lead to an elevation in nitric oxide synthesis. Previous studies have demonstrated that intestinal bacteria can secrete nitric oxide molecules, thereby altering the host’s own gene expression capabilities (42). Relevant reports have indicated the dual nature of NO in organisms. Under physiological conditions, low concentrations of NO are produced via the L-Arg and constitutive NOS pathways, playing roles in signal transduction, maintaining vascular tone, and other physiological functions. However, under pathological conditions, the continuous production of large amounts of NO through the activation of inducible NOS exhibits cytotoxic effects. An appropriate amount of NO exerts neuroprotective effects, but an excess of it has neurotoxic effects (43, 44). Our research results indicate that the elevated levels of *Bacteroidetes* MAGs in the gut of depressed patients show a positive correlation with an increase in nitric oxide synthase; this elevation may precipitate an increase in microbiota-derived nitric oxide, which could either inflict direct neurotoxicity or perturb neural homeostasis via indirect mechanisms, thereby intensifying the progression of depressive pathology (45). Further studies are required to reveal how the NO released by *Bacteroidetes* further aggravates the development of depression.

This study revealed differences in gut microbiota between individuals with depression and healthy populations, but it has the following limitations: the cohort size was relatively small, requiring validation through large-scale, multi-center studies and exploration of differences across populations, geographical regions, and disease progression stages. While metagenomic analysis suggested an association between microbial metabolism and depression, there is a lack of animal or *in vitro* experiments to validate the causal relationship. The healthy control group was primarily composed of students, exhibiting homogeneity in age and lifestyle; future research should incorporate more diverse populations to enhance the generalizability of findings. Although this study has unveiled a highly precise predictive panel, the direct or indirect associations of *Arthrobacter* sp.*_U41, Bacillus cereus, Campylobacter rectus*, and *Pasteurella dagmatis* with depression remain uncharted. Future research should endeavor to elucidate the relationships between these four species and depression, alongside their interactions with the *Bacteroides* species.

### Conclusion

In summary, our study reveals significant alterations in the composition and function of fecal microbiota in patients with depression compared to healthy individuals, with a significant enrichment of species belonging to *Bacteroides* in individuals with depression. Furthermore, the involvement of *Bacteroides* MAGs in the nitric oxide synthesis pathway is significantly upregulated in the gut of individuals with depression. Using the relative abundance of certain bacteria as a classification feature yielded precise classification results (average AUC value = 0.950), and similar results were also obtained in other models (SVM: average AUC value = 0.940 and LR: average AUC value = 0.920). Our findings suggest that *Bacteroides* species and the nitric oxide produced by fecal microbiota may serve as effective targets for the treatment of depression.

## Supplementary Material

Reviewer comments

## Data Availability

The metagenomic sequencing data presented in the study are submitted to the China National GeneBank (project number: CNP0006487). The custom scripts used for data analysis are provided as supplemental materials. A STORMS checklist is available at https://zenodo.org/records/14189203 (46).

## SUPPLEMENTAL MATERIAL

Fig. S1 spectrum.03153-24-s0001.pdf Gut microbiota composition analysis in mild depression people and healthy controls.

Fig S2 spectrum.03153-24-s0002.pdf Correlation between differential MAGs and SDS scores in patients with depression.

Fig. S3 spectrum.03153-24-s0003.pdf (a) Predictive performance of the random forest model across distinct subgroups (male versus female); (b) Comparative analysis of the random forest model's predictive efficacy with and without the inclusion of mild depression patients in the cohort (1,000 runs).

Tables S1 to S6 spectrum.03153-24-s0004.xlsx Table S1: The rank sum test results at the genus level. Table S2: Mean value of functional genes. Table S3: Result of chi-square test for functional genes in Fig. 2c. Table S4: Taxonomic classification of MAGs. Table S5: Taxonomic classification and LDA scores of differentially abundant MAGs. Table S6: Feature importance.

Supplemental material spectrum.03153-24-s0005.txt Metagenomic workflow in our study.

OPEN PEER REVIEW reviewer-comments.pdf An accounting of the reviewer comments and feedback.

ASM does not own the copyrights to Supplemental Material that may be linked to, or accessed through, an article. The authors have granted ASM a non-exclusive, world-wide license to publish the Supplemental Material files. Please contact the corresponding author directly for reuse.
