# Supplementary material for: Exploring the hypothetical role of Bacteroides species in depression progression: insights from metagenomic analysis
Source: Microbiol Spectr. 2026 Aug 11;14(9):e03153-24. doi: 10.1128/spectrum.03153-24 (PMC13532182; doi:10.1128/spectrum.03153-24)
Supplement: Supplementary file 1 [file reviewer-comments.pdf]

AMERICAN SOCIETY FOR MICROBIOLOGY | Microbiology Spectrum

Exploring the Hypothetical Role of *Bacteroides* Species in Depression Progression: Insights from Metagenomic Analysis

Zihua Li, Jingxi Sun, Jinlong Yang, Panpan Han, Liping Min, Yongkang Cheng, Yuanqiang Zou, and Zhuhua Liu

Corresponding Author(s): Zhuhua Liu, General Hospital of Ningxia Medical University

Review Timeline:

Submission Date:	December 9, 2024
Editorial Decision:	February 17, 2025
Revision Received:	April 21, 2025
Editorial Decision:	June 3, 2025
Revision Received:	August 1, 2025
Editorial Decision:	September 26, 2025
Revision Received:	November 17, 2025
Editorial Decision:	December 9, 2025
Revision Received:	February 4, 2026
Accepted:	February 28, 2026

Editor: Qi Su

Reviewer(s): Disclosure of reviewer identity is with reference to reviewer comments included in decision letter(s). The following individuals involved in review of your submission have agreed to reveal their identity: Jian Zhou (Reviewer #1); Inga Bazukyan (Reviewer #4)

Transaction Report:

(Note: With the exception of the correction of typographical or spelling errors that could be a source of ambiguity, letters and reports are not edited. The original formatting of letters and referee reports may not be reflected in this compilation.)

DOI: https://doi.org/10.1128/spectrum.03153-24

Re: Spectrum03153-24 (Exploring the Hypothetical Role of *Bacteroides* Species in Depression Progression: Insights from Metagenomic Analysis)

Dear Dr. Zhuhua Liu:

Thank you for the privilege of reviewing your work. Below you will find my comments, instructions from the Spectrum editorial office, and the reviewer comments.

Please return the manuscript within 60 days; if you cannot complete the modification within this time period, please contact me. If you do not wish to modify the manuscript and prefer to submit it to another journal, notify me immediately so that the manuscript may be formally withdrawn from consideration by Spectrum.

Revision Guidelines
To submit your modified manuscript, log into the submission site at https://spectrum.msubmit.net/cgi-bin/main.plex. Go to Author Tasks and click the appropriate manuscript title to begin. The information you entered when you first submitted the paper will be displayed; update this as necessary. Note the following requirements:

• Upload point-by-point responses to the issues raised by the reviewers in a file named "Response to Reviewers," NOT in your cover letter.
• Upload a compare copy of the manuscript (without figures) as a "Marked-Up Manuscript" file.
• Upload a clean .DOC/.DOCX version of the revised manuscript and remove the previous version.
• Each figure must be uploaded as a separate, editable, high-resolution file (TIFF or EPS preferred), and any multipanel figures must be assembled into one file.
• Any supplemental material intended for posting by ASM should be uploaded with their legends separate from the main manuscript. You can combine all supplemental material into one file (preferred) or split it into a maximum of 10 files with all associated legends included.

For complete guidelines on revision requirements, see our Submission and Review Process webpage. Submission of a paper that does not conform to guidelines may delay acceptance of your manuscript.

Data availability: ASM policy requires that data be available to the public upon online posting of the article, so please verify all links to sequence records, if present, and make sure that each number retrieves the full record of the data. If a new accession number is not linked or a link is broken, provide Spectrum production staff with the correct URL for the record. If the accession numbers for new data are not publicly accessible before the expected online posting of the article, publication may be delayed; please contact production staff (Spectrum@asmusa.org) immediately with the expected release date.

Publication Fees: For information on publication fees and which article types are subject to charges, visit our website. If your manuscript is accepted for publication and any fees apply, you will be contacted separately about payment during the production process; please follow the instructions in that e-mail. Arrangements for payment must be made before your article is published.

ASM Membership: Corresponding authors may join or renew ASM membership to obtain discounts on publication fees. Need to upgrade your membership level? Please contact Customer Service at Service@asmusa.org.

The ASM Journals program strives for constant improvement in our submission and publication process. Please tell us how we can improve your experience by taking this quick Author Survey.

Thank you for submitting your paper to Spectrum.

Sincerely,
Qi Su
Editor
Microbiology Spectrum

Reviewer #1 (Comments for the Author):

Overall Review
This study explores the role of gut microbiota in depression using shotgun metagenomic sequencing to compare 28 depression patients and 26 healthy individuals. Key findings include:
1. **Bacteroides enrichment**: Depression patients showed significant enrichment of *Bacteroides* species (e.g., *B.

xylanisolvens*, *B. caccae*, *B. stercoris*), which correlated with upregulated nitric oxide (NO) synthesis pathways.
2. **Functional dysbiosis**: Depression-associated microbiota exhibited metabolic shifts toward histamine, p-cresol, and NO synthesis, while healthy controls showed glutamate and SCFA-related pathways.
3. **Diagnostic potential**: A combination of five *Bacteroides* MAGs achieved high diagnostic accuracy for depression (AUC = 0.834) using a random forest model.
4. **Mechanistic insight**: Elevated *Bacteroides* abundance may drive NO overproduction, potentially contributing to neurotoxicity and depression pathology.

This work advances understanding of gut-brain axis mechanisms in depression and highlights microbial markers for diagnostics/therapeutics.

Paper Strengths
1. **Methodological rigor**: High-quality MAG reconstruction (>70% completeness) with multi-tool binning (MetaBAT2, MaxBin2), de-replication, and validation via GTDB-Tk.
2. **Functional insights**: Linking *Bacteroides* enrichment to nitric oxide synthase (K00491) via gene annotation and correlation analysis.
3. **Clinical relevance**: Identification of *Bacteroides*-based biomarkers with high predictive power (AUC >0.82).
4. **Innovative design**: Integration of metagenomic assembly, functional modules (e.g., gut-brain axis pathways), and machine learning for biomarker discovery.
5. **Detailed reporting**: Transparent workflows for sequencing, quality control, and statistical analysis (Wilcoxon test, PERMANOVA, randomForest).

Paper Weaknesses
1. **Limited sample size**: Small cohort (54 total subjects, 28 depressed), especially mild depression cases (*n*=3), reducing statistical power and generalizability.
2. **Causality gap**: Observational design cannot confirm if microbial changes *cause* depression or result from it (e.g., diet, medication, lifestyle confounders unaddressed).
3. **Mechanistic validation**: No experimental validation (e.g., animal models) to test *Bacteroides*-NO-depression causality.
4. **Technical limitations**:
- No raw sequencing data/markdown code availability.
- Incomplete MAG functional annotation (e.g., unresolved KEGG/COG roles for NO-related genes).
5. **Lack of comparator studies**: Limited comparison to prior depression microbiome studies (e.g., Jiang et al., 2015; Valles-Colomer et al., 2019) to contextualize *Bacteroides* findings.

Questions to Authors
1. **Confounding factors**: Did you control for diet, medication, or comorbidities that might influence microbiota composition?
2. **Causality**: Are there plans to validate findings in germ-free animal models colonized with depression-associated *Bacteroides* strains?
3. **Functional validation**: How does *Bacteroides*-derived NO directly interact with host neurons? Are there in vitro assays to test this?
4. **Diagnostic specificity**: Could the *Bacteroides* biomarkers distinguish depression from other psychiatric disorders (e.g., anxiety)?
5. **Technical reproducibility**: Will sequencing raw data and custom scripts be made publicly accessible?

Overall Score: **7/10**
Rationale: The study provides novel insights into gut microbiota's role in depression with robust metagenomic methodologies and clinically relevant biomarkers. However, small sample size, lack of mechanistic validation, and insufficient discussion of confounders limit immediate impact. Addressing these in revision (e.g., expanding cohorts, experimental validation) would strengthen significance.

Reviewer #2 (Comments for the Author):

This article explored the depression etiology from the point of view of metagenomic analysis of gut microbiota. The authors claimed that NO from gut bacteria is involved in depression. However, there is lack of direct evidence and the authors neither assayed NO in the gut nor cited reference in the Discussion to support their conclusion. It is reported that that gastrointestinal

microbiota can generate substantial amounts of NO by DNRA, so the pathway of DNRA may be investigated in this study. It is suggested to revise the Discussion to emphasize the novelty of this study.

Lines 34-36: There are typos in the Abstract.
Line 37: The author did not do enrichment of module/pathway, but the copies of the gene of an enzyme, which is suggested to do.
Line 39: The author did not assay the content of nitric oxide.
Line 86: 7 should be in superscript.
Lines 97-105: Can metagenomics identify each species or strain?
Line 111: There are articles studying gut microbiota in depression, please cite them.
Lines 175-179, 181-182, 184-186: It is not suggested to present the commands. Please state the main parameters in normal sentences. And the same comments for later commands.
Lines 213-232: misused marks, uncompleted sentences, grammar problems.
Line 170: 51 participants vs Line 258: 28 depression patients and 26 healthy 259 individuals, please check the number.
Lines 285-290: Please delete these sentences, because they are not the results.
Line 292: It is suggested to use the word "significant" instead of "notable", if appropriate.
Line 303: fig1b or fig2b?
Line 309: ware or were or was?
Lines 337-347; 357-363: Do not repeatedly list these species names in the text.
Lines 348-356; 378-388: Move them to the Discussion.
Line 375: no fig3c
Fig4b-d: the labels and units of axes are not clear.
The term "sp" followed a genus names is the abbreviation of species and should be written as "sp." and not in italic. Also, the "_" in "fragilis_A" should be deleted and "A" should not be in italic.
Lines 392-393: "mean value" is the mean of what?
It is not very clear how the randforest tests were performed. Did the author do randforest 1023 times?
The authors identified both species and MAGs of the microbiota, and which of them is better to represent the microbiota and depression? Why the profile of MAGs rather than of species were used to do randforest and prediction?
L553: Lack references.

Reviewer #3 (Comments for the Author):

This manuscript presents an interesting study that explores the potential role of gut microbiota, specifically the Bacteroides spp., in the pathogenesis of depression. The research employs shotgun metagenomic sequencing to study the gut microbiome at the strain level. The results highlight significant differences in the gut microbiome composition between patients with depression and healthy controls, with a particular focus on the enrichment of Bacteroides spp. in the depressed group and their association with nitric oxide synthesis pathways.

The findings provide some insights into the microbial biomarkers for depression. However, the study has several limitations, particularly in terms of method description and results interpretation. The authors should consider addressing these points to strengthen their findings and increase the potential impact of their research.

The suggestions for improvement are listed below:
Major points:
1. It is important to mention whether the sample size (28 depression patients and 26 healthy controls) was calculated to ensure statistical power. If not, the authors should acknowledge this limitation and suggest future studies with larger sample sizes to validate these findings.

2. The healthy control group consists of students, who may not completely represent the general population in terms of age, lifestyle, and mental health status. A more diverse control group, ideally matched in terms of age, gender, and lifestyle factors, would strengthen the conclusions of the study. This point should be clarified.

3. Whether the wording "fecal microbiome" is more appropriate than "gut microbiome", as it were the stools samples (Line148 Collect fecal samples from all participants....") that been collected and profiled?

4. whether any host reads were contained in the raw metagenomic data? And if so, how they were processed?

5. While the authors suggest that increased levels of Bacteroides might contribute to elevated nitric oxide synthesis, they do not provide direct evidence of the impact of nitric oxide on depression progression. The role of nitric oxide in the brain and its neurotoxic effects remain speculative. The authors should consider providing more in-depth analysis or references to support the claim that elevated nitric oxide is detrimental to the nervous system and contributes to depression.

6. Some other relevant pathways (e.g. "endocrine, immune, and neuroactive pathways" mentioned in Line85) and their potential

interactions that may also contribute to depression progression should be briefly mentioned, apart from the nitric oxide synthesis as a key metabolic pathway.

7. The authors used a random forest model to predict depression based on the microbiome. It would be helpful to see further validation of the model using external datasets or cross-validation methods to assess the stability and reproducibility of the findings.

8. The manuscript would benefit from a more careful review for clarity, as some sections are dense and may be challenging for non-experts to follow. For example, the description of the methods for data processing and statistical analysis is quite technical and lengthy (see Line175: descriptions for reads processing by fastp took 5~6 lines of words), and some parts could be simplified or clarified. Additionally, there are occasional minor grammatical errors and phrasing issues that should be addressed to improve readability.

Minor points:
Line34: "Notably, we found that the MAGs enriched in people with depression mostly belonged to *Bactendicating* a close link between Bacteroides abundance and the development of depression...*deroides, impression*" where *Bactendicating* should be *Bacteroides, indicating*; *deroides, impression* should be *depression*.

Line40: "Specifically, the mean relative abundance about the genomes of Bacteroides xylanisolvens, Bacteroides caccae, Bacteroides fragilis, Bacteroides stercoris and Bacteroides ovatus..." where the genus name "Bacteroides" should be abbreviated after first appearance.

Line190: "Functional analysis of fecal *microbiomev* was using gene set" where *microbiomev* should be *microbiome*

Line260: "We identified Bacteroides, Phocaeicola, Faecalibacterium, Agathobacter, Segatella, Blautia as the *most prevalent taxa* at the genus level in all individuals..." where description of *most prevalent taxa* is not precise, use exact percentage or portion instead. See also in Line272 (*lower*) and several other places.

Line264: why "Wilcoxon rank-sum" model was used to analyze variance? The same for "chi-square tests" (Line296).

Line281: it is not clear on what basis "samples from individuals with mild depression were excluded from the further study."? As this could be important for downstream predictive analysis by random forest model.

Line298 "with depression, while 654 genes showed significantly increased expression in the gut microbiota of healthy individuals..." could any these differential abundant genes be taxonomically assigned? And whether they could be identified to be included in any binned MAGs?

Line314, the decriptive parts of analyzing pipelines within the section 3.4 were mostly redundant with Material and Method.

Line335, the taxonomy names occupied too much texts, which ideally should be presented as table.

Review Report

Reviewer: Inga Bazukyan, PhD, Associate Professor, Yerevan State University, Faculty of Biology, Department of Biochemistry, Microbiology, and Biotechnology.

This is the review report for the research article **Spectrum03153-24**, titled "V*aginal Lactobacillus Postbiotics Ameliorate Gardnerella vaginalis-Induced Bacterial Vaginosis by Regulating Vaginal Microbiota and Restoring Th17/Treg Balance"*, authored by **Zihua Li, Jingxi Sun, Jinlong Yang, Panpan Han, Liping Min, Yongkang Cheng, Yuanqiang Zou, Zhuhua Liu.**

General Comments

The connection between the gut microbiome and depression is an active area of research, often referred to as the "gut-brain axis." This bidirectional communication system between the gut and the central nervous system involves neural, immune, endocrine, and metabolic pathways. Several key findings have already been established by various specialists, including changes in microbial composition (such as a decrease in the amount of Lactobacilli and Bifidobacteria), an increase in representatives of Enterobacteriaceae, and the fact that gut dysbiosis can increase intestinal permeability ("leaky gut"). This allows lipopolysaccharides (LPS) and other inflammatory molecules to enter the circulation, leading to higher levels of pro-inflammatory cytokines (e.g., IL-6, TNF-α), which are often observed in individuals with depression. Additionally, a decrease in the levels of short-chain fatty acid-producing bacteria has also been noted. As we can see, this topic is very interesting and relevant, though it is still not fully explored. The detection of multiple representatives of *Bacteroides*is insufficient. The article is poorly organized; this is evident even in the Abstract (lines 34-36), which contains multiple typos. Most importantly, the experimental groups of patients, aged 18-60, were compared with a control group consisting of senior and graduate students (who I don't believe can be close to 60). Age plays a key role in the gut microbiome, and aging significantly affects its composition.

Specific Comments & Suggestions

Introduction:

There are no references from lines 94 to 129, which constitutes about half of the introduction.

Methods:

1. Line 141 - the authors mention 25 patients with diagnosed depression, while the abstract states 28.
2. The male-to-female ratio was 27:25; is it the same for the control group?
3. There is no information about treatment with antidepressants (what kind of antidepressants?), which can also affect the gut microbiome. The same applies to the control group.
4. The fluctuation range of SDS scores for patients is 53.75-112.5, which is too broad and may impact the experiment.
5. Line 152 - The authors mentioned that the patients stopped using the drugs for two weeks or longer. However, how much longer, and how many patients stopped using the drugs for more than two weeks? Two weeks is not sufficient time for changes in the gut microbiome.
6. Line 170 - The authors mentioned that fecal samples were collected from 51 patients. However, in lines 141-154 and in the abstract, they refer to 54 patients. It is not important to mention 54 patients if some samples were excluded due to issues.

Results:

1. Line 248 - It would be more informative if the authors included the diet preferences of patients in Table 1.
2. Lines 303-313 - Metabolic activity in the gut microbiome can change for various reasons. Did the authors control for the diet or emotional status of the patients?
3. Line 322 - How did the authors calculate the percentage of contamination?
4. Line 336 - Typo: "Enrichment" should be corrected to "enrichment."
5. Lines 337-347, 357-363, and 402-425 - The authors listed the Latin names of bacteria, which is very difficult to read. It would be more helpful to see reasonable grouping or explanations.

Discussion:

It would be more interesting to compare the data obtained by this group with referenced data from other countries to detect any national differences. There are many reasons for changes in microbiome composition, particularly regarding the metabolic activity of bacteria.

Conclusion

Addressing the above points: particularly by including a discussion that compares the study’s findings with existing literature and clarifying the experimental groups, especially the age of

patients and other factors affecting them, suggests that **this article is not suitable for publication**.

Dear Editors and Reviewers:

We sincerely appreciate the valuable feedback provided by the editors and reviewers, whose insightful comments have significantly improved the quality of our manuscript. To facilitate the review process, all modifications made in response to these suggestions have been highlighted in blue text throughout the marked-Up manuscript.

Reviewer #1 (Comments for the Author):

Overall Review

This study explores the role of gut microbiota in depression using shotgun metagenomic sequencing to compare 28 depression patients and 26 healthy individuals. Key findings include:

1. **Bacteroides enrichment**: Depression patients showed significant enrichment of *Bacteroides* species (e.g., *B. xylanisolvens*, *B. caccae*, *B. stercoris*), which correlated with upregulated nitric oxide (NO) synthesis pathways.

2. **Functional dysbiosis**: Depression-associated microbiota exhibited metabolic shifts toward histamine, p-cresol, and NO synthesis, while healthy controls showed glutamate and SCFA-related pathways.

3. **Diagnostic potential**: A combination of five *Bacteroides* MAGs achieved high diagnostic accuracy for depression (AUC = 0.834) using a random forest model.

4. **Mechanistic insight**: Elevated *Bacteroides* abundance may drive NO overproduction, potentially contributing to neurotoxicity and depression pathology.

This work advances understanding of gut-brain axis mechanisms in depression and highlights microbial markers for diagnostics/therapeutics.

Paper Strengths

1. **Methodological rigor**: High-quality MAG reconstruction (>70% completeness) with multi-tool binning (MetaBAT2, MaxBin2), de-replication, and validation via GTDB-Tk.

2. **Functional insights**: Linking *Bacteroides* enrichment to nitric oxide synthase (K00491) via gene annotation and correlation analysis.

3. **Clinical relevance**: Identification of *Bacteroides*-based biomarkers with high predictive power (AUC >0.82).

4. **Innovative design**: Integration of metagenomic assembly, functional modules (e.g., gut-brain axis pathways), and machine learning for biomarker discovery.

5. **Detailed reporting**: Transparent workflows for sequencing, quality control, and statistical analysis (Wilcoxon test, PERMANOVA, randomForest).

Paper Weaknesses

1. **Limited sample size**: Small cohort (54 total subjects, 28 depressed), especially mild depression cases (*n*=3), reducing statistical power and generalizability.

2. **Causality gap**: Observational design cannot confirm if microbial changes *cause* depression or result from it (e.g., diet, medication, lifestyle confounders unaddressed).

3. **Mechanistic validation**: No experimental validation (e.g., animal models) to test *Bacteroides*-NO-depression causality.

4. **Technical limitations**:

- No raw sequencing data/markdown code availability.

- Incomplete MAG functional annotation (e.g., unresolved KEGG/COG roles for NO-related genes).

5. **Lack of comparator studies**: Limited comparison to prior depression microbiome studies (e.g., Jiang et al., 2015; Valles-Colomer et al., 2019) to contextualize *Bacteroides* findings.

Questions to Authors

1. **Confounding factors**: Did you control for diet, medication, or comorbidities that might influence microbiota composition?

Author response: Thank you for your question. The control group and the case group did not implement any dietary restrictions and both followed a free diet pattern. As for the medication, we have addressed this in the inclusion and exclusion criteria. Outpatient patients with first-time onset who had not taken any psychiatric medications or had discontinued them for two weeks or more prior to enrollment, and had no other underlying diseases, were included.

2. **Causality**: Are there plans to validate findings in germ-free animal models colonized with depression-associated *Bacteroides* strains?

Author response: Thank you for your review comments. Regarding whether we plan to validate our findings in a germ-free animal model with the colonization of depression-related Bacteroides strains, we are indeed considering this direction. We plan to use germ-free animal models in subsequent studies to verify the role of these depression-related *Bacteroides* strains in gut colonization and depression-related behaviors, in order to further confirm their potential impact on depression. We greatly appreciate your suggestion, and this validation will help strengthen our research findings.

3. **Functional validation**: How does *Bacteroides*-derived NO directly interact with host neurons? Are there in vitro assays to test this?

Author response: Thank you for your question. This study was conducted in a population, and we identified the nitric oxide synthase (NOS) gene in *Bacteroides*. Based on the literature, we know that NO may affect primary neurons. However, we have not yet

validated this conclusion in animal experiments; it is merely our hypothesis. In the future, we will design relevant animal experiments to verify this.

4. **Diagnostic specificity**: Could the *Bacteroides* biomarkers distinguish depression from other psychiatric disorders (e.g., anxiety)?

Author response: Thank you for your question. Our current sample only includes testing on the general population and individuals with depression, without including individuals with anxiety disorder. Your suggestion is excellent, and we plan to conduct a comparative study involving the general population, individuals with depression, and individuals with anxiety in the future, in order to further improve our research.

5. **Technical reproducibility**: Will sequencing raw data and custom scripts be made publicly accessible?

Author response: Thank you for your review comments. We confirm that all raw sequencing data and custom scripts are publicly accessible. The raw sequencing data have been deposited in the China National GeneBank DataBase (CNGBdb) under project accession number CNP0006487 (https://db.cngb.org/). The custom scripts used for data analysis are provided as Supplementary Materials accompanying this manuscript. All these contents are indicated in the DATA AVAILABILITY of the manuscript.

Overall Score: **7/10**

Rationale: The study provides novel insights into gut microbiota's role in depression with robust metagenomic methodologies and clinically relevant biomarkers. However, small sample size, lack of mechanistic validation, and insufficient discussion of confounders limit immediate impact. Addressing these in revision (e.g., expanding cohorts, experimental validation) would strengthen significance.

Reviewer #2 (Comments for the Author):

This article explored the depression etiology from the point of view of metagenomic analysis of gut microbiota. The authors claimed that NO from gut bacteria is involved in depression. However, there is lack of direct evidence and the authors neither assayed NO in the gut nor cited reference in the Discussion to support their conclusion. It is reported that that gastrointestinal microbiota can generate substantial amounts of NO by DNRA, so the pathway of DNRA may be investigated in this study. It is suggested to revise the Discussion to emphasize the novelty of this study.

Lines 34-36: There are typos in the Abstract.

Author response: Thanks for your suggestion. We have revised the Abstract.

Line 37: The author did not do enrichment of module/pathway, but the copies of the gene of an enzyme, which is suggested to do.

Author response: Thank you for your valuable feedback. We have supplemented this analysis of the section, and the results are now presented in Figure 2b. Additionally, the results and discussion sections have been accordingly updated to reflect this information (Line 305-319, Line 431-440).

Line 39: The author did not assay the content of nitric oxide.

Author response: Thank you for your valuable comment. As you pointed out, we did not test for NO, which is a flaw in our study. Due to the limited number of fecal samples from donors obtained in the earlier stage and the fact that NO is a gaseous molecule that easily evaporates, we did not attempt to detect NO in this study. In the future, we may consider conducting specific animal experiments to measure NO levels, and we will try to capture NO molecules using in situ probes.

Line 86: 7 should be in superscript.

Author response: Thanks. Done.

Lines 97-105: Can metagenomics identify each species or strain?

Author response: Thank you for your question. Metagenomics can indeed identify species or strains, primarily depending on the average nucleotide identity (ANI) among metagenome-assembled genomes (MAGs). A threshold of 95% is typically used to define bacterial species, while a threshold of 97% is employed for strain identification[1].

Reference: [1] Goris J, Konstantinidis K T, Klappenbach J A, et al. DNA–DNA hybridization values and their relationship to whole-genome sequence similarities [J]. International Journal of Systematic and Evolutionary Microbiology, 2007, 57(1): 81-91.

Line 111: There are articles studying gut microbiota in depression, please cite them.

Author response: Thanks for your suggestion. References have been added according to your recommendations.

Lines 175-179, 181-182, 184-186: It is not suggested to present the commands. Please state the main parameters in normal sentences. And the same comments for later commands.

Author response: Thank you for your valuable comment. We have made the necessary revisions(Line 179-217).

Lines 213-232: misused marks, uncompleted sentences, grammar problems.

Author response: Thank you for your valuable comment. We have done the revision.

Line 170: 51 participants vs Line 258: 28 depression patients and 26 healthy 259 individuals, please check the number.

Author response: Thank you for your valuable comment. We have made the necessary revisions, and the total number of individuals is 54.

Lines 285-290: Please delete these sentences, because they are not the results.

Author response: Thanks for your suggestion. This section has been deleted.

Line 292: It is suggested to use the word "significant" instead of "notable", if appropriate.

Author response: Thanks for your suggestion. The word "significant" has been used instead of "notable."

Line 303: fig1b or fig2b?

Author response: Thank you for your careful review. This error has been corrected.

Line 309: ware or were or was?

Author response: Thanks. "ware" should be "were".

Lines 337-347; 357-363: Do not repeatedly list these species names in the text.

Author response: We sincerely appreciate this suggestion. As recommended, we have removed all repeated species name listings from the main text and consolidated the complete taxonomic information into Supplementary Table 2.

Lines 348-356; 378-388: Move them to the Discussion.

Author response: Thanks. Following your suggestion, we have moved this section to the discussion part (Line 495-501).

Line 375: no fig3c

Author response: Thank you for your careful review. " fig3c " should be " fig4c ".

Fig4b-d: the labels and units of axes are not clear.

Author response: Thanks. Done.

The term "sp" followed a genus names is the abbreviation of species and should be written as "sp." and not in italic. Also, the "_" in "fragilis_A" should be deleted and "A" should not be in italic.

Author response: Thanks. Done.

Lines 392-393: "mean value" is the mean of what?

Author response: We sincerely appreciate the your insightful comment. Upon careful re-examination of section about construction of random forest model, we have recognized that the description lacked clarity. To improve readability and transparency, we have revised the methodology about random forest test procedure. The detailed procedure is now clearly

documented in the revised manuscript (Lines 213–232) and the corresponding results are also revised in Lines 403–417 of the manuscript. We apologize for any confusion caused by the initial wording and hope the revised text addresses this concern comprehensively.

It is not very clear how the randforest tests were performed. Did the author do randforest 1023 times?

Author response: We thank you for highlighting this important technical point. The full experimental workflow has now been systematically described in the Methods section (Lines 213–232). Meanwhile, The random forest analysis was run 1,000 times as now explicitly stated in Line 231 of the revised manuscript. We regret any confusion caused by prior ambiguous phrasing and have carefully verified this number.

The authors identified both species and MAGs of the microbiota, and which of them is better to represent the microbiota and depression? Why the profile of MAGs rather than of species were used to do randforest and prediction?

Author response: We appreciate your insightful comment. While species-level profiling provides more comprehensive taxonomic representation, we initially chose MAGs because they allow direct linkage between functional genes and specific bacteria, which is valuable for investigating depression-associated mechanisms. However, upon careful reconsideration, we agree that species-level profiles are indeed more appropriate for random forest prediction, as MAGs may lose partial genomic information. We have updated our analyses using species-level data, as described in Methods 2.5 and Results 3.5.

L553: Lack references.

Author response: Thanks for your suggestion. Following your suggestion, we have added the references.

Reviewer #3 (Comments for the Author):

This manuscript presents an interesting study that explores the potential role of gut microbiota, specifically the Bacteroides spp., in the pathogenesis of depression. The research employs shotgun metagenomic sequencing to study the gut microbiome at the strain level. The results highlight significant differences in the gut microbiome composition between patients with depression and healthy controls, with a particular focus on the enrichment of Bacteroides spp. in the depressed group and their association with nitric oxide synthesis pathways.

The findings provide some insights into the microbial biomarkers for depression. However, the study has several limitations, particularly in terms of method description and

results interpretation. The authors should consider addressing these points to strengthen their findings and increase the potential impact of their research.

The suggestions for improvement are listed below:

Major points:

1. It is important to mention whether the sample size (28 depression patients and 26 healthy controls) was calculated to ensure statistical power. If not, the authors should acknowledge this limitation and suggest future studies with larger sample sizes to validate these findings.

Author response: Thanks for your suggestion. Due to the limited number of patients, we were unable to collect a sufficient sample size. As you pointed out, the small sample size is indeed a limitation of our study. We have matched the corresponding healthy individuals based on the number of patients. In the future, we plan to expand the sample size and validate our findings in a larger population.

2. The healthy control group consists of students, who may not completely represent the general population in terms of age, lifestyle, and mental health status. A more diverse control group, ideally matched in terms of age, gender, and lifestyle factors, would strengthen the conclusions of the study. This point should be clarified.

Author response: Thanks for your suggestion. You mentioned that using students as the control group might limit the generalizability of the study conclusions to broader populations due to differences in age, lifestyle, and baseline mental health status. We fully acknowledge this limitation. The selection of students as controls in this study was primarily based on operational feasibility and ease of sample accessibility, as obtaining heterogeneous control samples could have been challenging. We have explicitly addressed this issue in the revised manuscript's Discussion section and explored its potential implications for result interpretation. In future large-scale population validation studies, we plan to recruit more heterogeneous control groups, rigorously matched for age, gender, occupational status, and lifestyle factors (e.g., physical activity, sleep patterns), to better represent the general population.

3. Whether the wording "fecal microbiome" is more appropriate than "gut microbiome", as it were the stools samples (Line148 Collect fecal samples from all participants....") that been collected and profiled?

Author response: Thank you for your valuable feedback. The issue you raised regarding the use of "fecal microbiome" and "gut microbiome" is very reasonable. Indeed, considering that our samples are directly obtained from feces (as mentioned in line 148: "fecal samples were collected from all participants..."), using "fecal microbiome" may be more accurate. We will revise the relevant terminology in the paper based on your suggestion to ensure greater precision.

4. whether any host reads were contained in the raw metagenomic data? And if so, how they were processed?

Author response: Thanks for your question. We confirm that host-derived reads were present in the raw metagenomic data, as is commonly observed due to unavoidable host tissue contact during sampling. These reads were effectively removed during our bioinformatic processing using Bowtie2 alignment against the human reference genome (GRCh38), as detailed in Methods section 2.2.

5. While the authors suggest that increased levels of Bacteroides might contribute to elevated nitric oxide synthesis, they do not provide direct evidence of the impact of nitric oxide on depression progression. The role of nitric oxide in the brain and its neurotoxic effects remain speculative. The authors should consider providing more in-depth analysis or references to support the claim that elevated nitric oxide is detrimental to the nervous system and contributes to depression.

Author response: Thanks for your suggestion. As you pointed out, we did not measure NO, which is a limitation of our study. Due to the limited number of donor fecal samples collected in the early stages and the fact that NO is a gaseous molecule that easily evaporates, we did not attempt to measure NO in this study. In the future, we may consider conducting specific animal experiments to measure NO levels, and we will try to use in situ probes to capture NO molecules. Additionally, we have added new references to the paper.

6. Some other relevant pathways (e.g. "endocrine, immune, and neuroactive pathways" mentioned in Line85) and their potential interactions that may also contribute to depression progression should be briefly mentioned, apart from the nitric oxide synthesis as a key metabolic pathway.

Author response: We thank you for this valuable suggestion. We have now supplemented the discussion with additional details about these pathways in the revised manuscript (Line 88-99).

7. The authors used a random forest model to predict depression based on the microbiome. It would be helpful to see further validation of the model using external datasets or cross-validation methods to assess the stability and reproducibility of the findings.

Author response: We appreciate the your constructive suggestion regarding model validation. In our study, we have already implemented validation approaches to evaluate model performance, including:(1) Five-fold cross-validation to assess predictive accuracy;(2) Repeated random splitting of the dataset into training and validation sets (1000 iterations) to evaluate model stability. These validation procedures are described in detail in the Methods section (Section 2.5).

8. The manuscript would benefit from a more careful review for clarity, as some sections are dense and may be challenging for non-experts to follow. For example, the description of the methods for data processing and statistical analysis is quite technical and lengthy (see Line175: descriptions for reads processing by fastp took 5~6 lines of words), and some parts

could be simplified or clarified. Additionally, there are occasional minor grammatical errors and phrasing issues that should be addressed to improve readability.

Author response: We sincerely appreciate the your valuable suggestions to improve the manuscript's clarity and accessibility. In response to these comments, we have simplified overly technical descriptions, improved logical flow and reduced redundancy in methodological details. Meanwhile, we have corrected grammatical errors, improved sentence structure and enhanced overall readability.

Minor points:

Line34: "Notably, we found that the MAGs enriched in people with depression mostly belonged to *Bactendicating* a close link between Bacteroides abundance and the development of depression...*deroides, impression*" where *Bactendicating* should be *Bacteroides, indicating*; *deroides, impression* should be *depression*.

Author response: Thanks. Done.

Line40: "Specifically, the mean relative abundance about the genomes of Bacteroides xylanisolvens, Bacteroides caccae, Bacteroides fragilis, Bacteroides stercoris and Bacteroides ovatus..." where the genus name "Bacteroides" should be abbreviated after first appearance.

Author response: Thanks. We have made the revisions as per your suggestion and ensured that the genus name "Bacteroides" is abbreviated after its first appearance.

Line190: "Functional analysis of fecal *microbiomev* was using gene set" where *microbiomev* should be *microbiome*

Author response: Thanks. Done.

Line260: "We identified Bacteroides, Phocaeicola, Faecalibacterium, Agathobacter, Segatella, Blautia as the *most prevalent taxa* at the genus level in all individuals..." where description of *most prevalent taxa* is not precise, use exact percentage or portion instead. See also in Line272 (*lower*) and several other places.

Author response: Thanks for your valuable comments, we have added specific percentages and specific significance P-values to your mentions to complete our description

Line264: why "Wilcoxon rank-sum" model was used to analyze variance? The same for "chi-square tests" (Line296).

Author response: Thanks for your valuable comments, we used the Wilcoxon rank-sum test for microbial taxa (relative abundance data) because it is robust for non-normally distributed, sparse data common in microbiome studies. For the differential gene analysis, we employed chi-square tests since we focused on identifying whether specific genes were associated with depression.

Line281: it is not clear on what basis "samples from individuals with mild depression were excluded from the further study."? As this could be important for downstream predictive analysis by random forest model.

Author response: We thank you for raising this important point. The exclusion was based on two key observations: (1) The three mild depression cases showed SDS scores averaging 56.25 (clinically borderline, as scores ≤53 are typically considered non-depressed); (2) Preliminary analyses revealed no significant microbiota differences between these cases and healthy controls (SFig1). We excluded these borderline cases to ensure clearer separation between depressed and non-depressed groups in our predictive modeling. This decision was made post hoc after verifying their lack of microbial differentiation, not a priori. We have now clarified this rationale in the revised result section (Lines 283-288).

Line298 "with depression, while 654 genes showed significantly increased expression in the gut microbiota of healthy individuals..." could any these differential abundant genes be taxonomically assigned? And whether they could be identified to be included in any binned MAGs?

Author response: We appreciate your insightful question. While only a portion of these differentially abundant genes could be taxonomically assigned due to inherent limitations in metagenomic binning and gene catalog construction, we successfully linked many functional genes to specific bacteria through MAG functional analysis. Specifically, by functionally annotating the differentially abundant MAGs (Fig4a) and comparing with the gene catalog (Fig2c), we identified overlapping functional features that enabled us to associate key microbial functions with specific bacterial taxa.

Line314, the decriptive parts of analyzing pipelines within the section 3.4 were mostly redundant with Material and Method.

Author response: Thanks for your suggestion, we have revised this part in the manuscript

Line335, the taxonomy names occupied too much texts, which ideally should be presented as table.

Author response: We thank your for this constructive suggestion. We have now moved the complete taxonomic information into a structured Supplementary Table 2, which provides the full list of MAGs with their taxonomic classifications and LDA scores.

Re: Spectrum03153-24R1 (**Exploring the Hypothetical Role of *Bacteroides* Species in Depression Progression: Insights from Metagenomic Analysis**)

Dear Dr. Zhuhua Liu:

Thank you for the privilege of reviewing your work. Below you will find my comments, instructions from the Spectrum editorial office, and the reviewer comments.

Please return the manuscript within 60 days; if you cannot complete the modification within this time period, please contact me. If you do not wish to modify the manuscript and prefer to submit it to another journal, notify me immediately so that the manuscript may be formally withdrawn from consideration by Spectrum.

Revision Guidelines
To submit your modified manuscript, log into the submission site at https://spectrum.msubmit.net/cgi-bin/main.plex. Go to Author Tasks and click the appropriate manuscript title to begin. The information you entered when you first submitted the paper will be displayed; update this as necessary. Note the following requirements:

• Upload point-by-point responses to the issues raised by the reviewers in a file named "Response to Reviewers," NOT in your cover letter.
• Upload a compare copy of the manuscript (without figures) as a "Marked-Up Manuscript" file.
• Upload a clean .DOC/.DOCX version of the revised manuscript and remove the previous version.
• Each figure must be uploaded as a separate, editable, high-resolution file (TIFF or EPS preferred), and any multipanel figures must be assembled into one file.
• Any supplemental material intended for posting by ASM should be uploaded with their legends separate from the main manuscript. You can combine all supplemental material into one file (preferred) or split it into a maximum of 10 files with all associated legends included.

For complete guidelines on revision requirements, see our Submission and Review Process webpage. Submission of a paper that does not conform to guidelines may delay acceptance of your manuscript.

Data availability: ASM policy requires that data be available to the public upon online posting of the article, so please verify all links to sequence records, if present, and make sure that each number retrieves the full record of the data. If a new accession number is not linked or a link is broken, provide Spectrum production staff with the correct URL for the record. If the accession numbers for new data are not publicly accessible before the expected online posting of the article, publication may be delayed; please contact production staff (Spectrum@asmusa.org) immediately with the expected release date.

Publication Fees: For information on publication fees and which article types are subject to charges, visit our website. If your manuscript is accepted for publication and any fees apply, you will be contacted separately about payment during the production process; please follow the instructions in that e-mail. Arrangements for payment must be made before your article is published.

ASM Membership: Corresponding authors may join or renew ASM membership to obtain discounts on publication fees. Need to upgrade your membership level? Please contact Customer Service at Service@asmusa.org.

The ASM Journals program strives for constant improvement in our submission and publication process. Please tell us how we can improve your experience by taking this quick Author Survey.

Thank you for submitting your paper to Spectrum.

Sincerely,
Qi Su
Editor
Microbiology Spectrum

Reviewer #1 (Comments for the Author):

Major Concerns
Inadequate Sample Size and Homogeneous Control Group
The study includes only 28 depression patients (3 mild, 25 major) and 26 healthy controls, primarily students. While the authors acknowledge the small sample size, no formal power analysis is provided, and the exclusion of mild depression cases (based on

borderline SDS scores and nonsignificant microbiota differences) lacks sensitivity analysis to assess its impact on biomarker stability. This undermines the generalizability of findings, especially for subgroup analyses (e.g., mild vs. major depression).
The control group's homogeneity (similar age, lifestyle, and environmental factors) introduces potential bias, as confounding variables like diet, stress, or physical activity are not controlled for or reported. The reliance on student volunteers limits external validity, as the general population may exhibit different microbiota profiles.
Suggestion: Report sample size justification, conduct sensitivity analyses excluding mild cases, and explicitly describe control group demographics (e.g., diet, medication history) to address confounding.
Lack of Causal Validation and Functional Mechanistic Evidence
The study establishes an association between Bacteroides enrichment and nitric oxide (NO) synthesis pathways but provides no experimental validation of causality. Key missing evidence includes:
Direct measurement of NO levels in fecal/serum samples or brain tissue of patients.
In vitro/in vivo experiments demonstrating Bacteroides-derived NO impacts neuronal function (e.g., cell viability, neurotransmitter signaling).
Germ-free animal models colonized with depression-associated Bacteroides strains to assess behavioral and neurobiological outcomes.
The mechanistic link between gut-derived NO and depression pathogenesis remains speculative. While NO's neurotoxic potential is mentioned, the pathway by which gut-produced NO influences the brain (e.g., blood-brain barrier permeability, immune-mediated signaling) is not experimentally or theoretically elaborated.
Suggestion: Supplement with pilot data on NO concentration measurements or cite preclinical studies supporting gut-brain NO signaling. Clearly distinguish between correlative findings and hypothesized mechanisms.
Methodological Rigor in Statistical and Microbiome Analysis
The use of chi-square tests for differential gene analysis and Wilcoxon rank-sum tests for taxa abundance lacks transparency regarding multiple testing correction (e.g., FDR adjustment), critical for high-dimensional microbiome data to avoid false positives.
While 350 metagenome-assembled genomes (MAGs) are generated, species-level annotation for Bacteroides MAGs is incomplete (e.g., "Bacteroides sp."), and functional annotation of NO synthase (K00491) does not confirm strain-specific expression. The random forest model's high AUC (0.950) is not benchmarked against alternative algorithms (e.g., SVM, logistic regression) or clinical covariates, limiting its interpretability as a standalone biomarker.
Suggestion: Report FDR-corrected p-values for differential analyses, refine MAG taxonomy to the species level where possible, and include comparative model performance metrics.
Incomplete Results Presentation and Limited Literature Integration
Key visualizations (e.g., Fig4b-d) lack clarity: axes labels for gene abundance are undefined, and confidence intervals for correlations are absent, hindering reproducibility. The absence of associations between SDS scores and Bacteroides abundance (or other biomarkers) weakens clinical relevance.
The discussion underutilizes comparisons with prior depression microbiome studies (e.g., Jiang et al., 2015; Valles-Colomer et al., 2019), particularly regarding the Bacteroidetes/Firmicutes ratio or SCFA-producing bacteria like Faecalibacterium prausnitzii. Mechanistic connections to immune-inflammatory pathways (e.g., IL-6, TNF-α) are cursorily mentioned but not linked to observed microbiota changes.
Suggestion: Add scatter plots of SDS scores vs. microbial abundances, integrate comparative data from prior studies, and elaborate on immune-microbiota interactions.
Minor Concerns
Data Availability and Reproducibility
While raw data and scripts are deposited in CNGBdb, the manuscript does not provide a direct hyperlink to the dataset, and technical details (e.g., programming languages, package versions for custom scripts) are missing, complicating reproducibility.
Taxonomic naming inconsistencies (e.g., "Arthrobacter sp U41" instead of "Arthrobacter sp.") and undefined abbreviations (e.g., "MAGs" without full expansion in first mention) hinder clarity.
Technical and Editorial Precision
Fig2c and Fig4a lack clear mapping between microbial taxa and metabolic modules (e.g., which Bacteroides species contribute to NO synthesis).
Grammatical errors and awkward phrasing persist (e.g., "ware significant difference" instead of "were significant differences"), requiring a thorough language edit.
Overall Assessment
This study offers valuable insights into the gut microbiome's role in depression through robust metagenomic methods and identifies promising biomarkers. However, critical limitations-including small sample size, lack of causal validation, and incomplete mechanistic characterization-restrict the strength of its conclusions. Addressing these concerns would solidify the study's contribution to understanding depression pathogenesis and advancing microbial biomarker research.
Recommendation: Revise with experimental validation, expanded sample size, and clearer methodological transparency to strengthen causal inference and reproducibility.

Reviewer #2 (Comments for the Author):

Improvements have been made, however the ms has not meet the level to publish.

Sigla such as MAG, HC and MDD should be presented in full forms at its first appearance in the text and legends.
Why genus-level data, rather than species-level data, were used for alpha and beta diversity analysis?
Lines 145-147: 25+26<>54, 25+27<>54
Lines197-199: It seems authors using LDA to identify the bacterial abundance, rather than the expressions of genes (Fig. 3). In Fig.2b, it is pathways but not genes. It is not accurate to use the term "expressed genes" , since the authors used DNA sequence but not RNA sequence.
Lines 269,277: Both the statistics of Wilcoxon and P value should be reported the the text, and use the word "significant" instead of "notable".
Fig.2: Wrong legends. There are no Shannon index and Simpson index. What is MDD?
Line 306: How was the pathway enrichment performed? The methods seems not be presented in the Methods part. Althogth the authors mentioned EggNOG, it was not clear.
Fig3: There are typos in the legends. What are Bacillota A and C?
Fig4b-d: the labels and units of axes are not clear. What do the values of x and y axis represent? Bacteroides is not one of the 4 markers from Randforest, so what is the sense to do this analysis?
Line 371: In the response file the authors said that they used species-level data to do randomforest analysis, however in this sentence they used MAGs.
Line 373: It seems importance score to select the optimal features. So, the importance scores should be reported in the text.
In the disscussion, the authors disscussed Bacteroides and Faecalibacterium instead of the 4 markers, why? What is the purpose of this study?

SFig1b: It seems there are only three MD points. What do digits 0.56 and 0.28 reprenst? Since you excluded 3 MDs, it is better to state that 25 depression patients and 26 healthy microbiota were used in the Methods part, and perform all analyses using the 51 microboita.

Reviewer #3 (Comments for the Author):

Many thanks to the authors for their time and efforts putting into this revision. I do not have further comments.

Dear Editors and Reviewers:

We sincerely appreciate the valuable feedback provided by the editors and reviewers, whose insightful comments have significantly improved the quality of our manuscript. To facilitate the review process, all modifications made in response to these suggestions have been highlighted in blue text throughout the marked-Up manuscript.

Reviewer #1 (Comments for the Author):

Major Concerns

1:Inadequate Sample Size and Homogeneous Control Group

The study includes only 28 depression patients (3 mild, 25 major) and 26 healthy controls, primarily students. While the authors acknowledge the small sample size, no formal power analysis is provided, and the exclusion of mild depression cases (based on borderline SDS scores and nonsignificant microbiota differences) lacks sensitivity analysis to assess its impact on biomarker stability. This undermines the generalizability of findings, especially for subgroup analyses (e.g., mild vs. major depression).

The control group's homogeneity (similar age, lifestyle, and environmental factors) introduces potential bias, as confounding variables like diet, stress, or physical activity are not controlled for or reported. The reliance on student volunteers limits external validity, as the general population may exhibit different microbiota profiles.

Suggestion: Report sample size justification, conduct sensitivity analyses excluding mild cases, and explicitly describe control group demographics (e.g., diet, medication history) to address confounding.

Response to Comment on Sample Size and Control Group Homogeneity

A. Sample Size Justification

We acknowledge the limited sample size in this exploratory study (n=54total participants). As stated in the Methodssection (2.1), recruitment was constrained by strict inclusion/exclusion criteria (e.g., first-onset depression, no psychotropic medication for ⩾2 weeks, exclusion of comorbidities). These criteria ensured cohort homogeneity but reduced sample availability. While formal power analysis was not performed, our metagenomic approach generated high-resolution data (350 non-redundant MAGs, 1.7M genes) with statistically significant differences (e.g., p<0.05for Bacteroidota/Bacillota shifts; Fig1b-c). The discriminatory power of 4 species (AUC=0.920; Fig5) further supports the robustness of findings within this cohort.

B. Exclusion of Mild Depression Cases

Exclusion of mild depression cases (n=3) was based on:

Statistical Non-significance: Microbiota composition in mild depression showed no significant differences vs. controls (Shannon/Simpson indices, β-diversity; Sfig1), potentially diluting group contrasts.

Clinical Threshold: SDS scores (53.75 – 56.25) were borderline for depression diagnosis (SDS>53 required; Methods 2.1), aligning with diagnostic rigor.No

sensitivity analysis was performed due to the minimal subgroup size (n=3), which lacks statistical power. Future studies with larger mild-depression cohorts will address this.

C. Control Group Homogeneity

The control group (n=26students) was intentionally selected for:

Reducing Confounders: Similar age/lifestyle minimized variability from diet, environment, or medication (all controls screened for no psychiatric history, drug use, or chronic diseases; Methods 2.1).

Feasibility: Access to longitudinal samples from matched demographics was prioritized for initial biomarker discovery. We agree external validity requires broader populations and have noted this limitation (Discussion).

2:Lack of Causal Validation and Functional Mechanistic Evidence

The study establishes an association between Bacteroides enrichment and nitric oxide (NO) synthesis pathways but provides no experimental validation of causality. Key missing evidence includes:

Direct measurement of NO levels in fecal/serum samples or brain tissue of patients.

In vitro/in vivo experiments demonstrating Bacteroides-derived NO impacts neuronal function (e.g., cell viability, neurotransmitter signaling).

Germ-free animal models colonized with depression-associated Bacteroides strains to assess behavioral and neurobiological outcomes.

The mechanistic link between gut-derived NO and depression pathogenesis remains speculative. While NO's neurotoxic potential is mentioned, the pathway by which gut-produced NO influences the brain (e.g., blood-brain barrier permeability, immune-mediated signaling) is not experimentally or theoretically elaborated.

Suggestion: Supplement with pilot data on NO concentration measurements or cite preclinical studies supporting gut-brain NO signaling. Clearly distinguish between correlative findings and hypothesized mechanisms.

Response to Comment on Causal Validation and Mechanistic Evidence

A: NO Synthesis Pathway Association

Our study focused on identifying associations between *Bacteroides* enrichment and NO synthesis, not causality. Key evidence includes:

Metagenomic Linkage: All *Bacteroides* MAGs enriched in depression were annotated with nitric oxide synthase (K00491; Fig4a).

Linear Correlation: Positive correlation between *Bacteroides* MAG abundance (e.g., *B. stercoris, B. thetaiotaomicron*) and K00491 gene levels ($p<0.05$; Fig4b-d).

Functional Module Enrichment: The NO synthesis pathway was significantly upregulated in depression vs. controls (Fig2b).

B: Mechanistic Plausibility

While we did not measure fecal/serum NO or conduct animal experiments, the mechanism is supported by:

Literature Evidence: NO exhibits neurotoxic effects at high concentrations (Discussion, lines 549 − 553), citing:

Moncada et al. (1991): NO's dual role in neuroprotection/toxicity.

Bender & Beavo (2004): NO-mediated neuronal signaling pathways.

C: Clarification of Correlative vs. Causal Claims

We emphasize our conclusions as hypothetical:

"This study shed light on the potential role of gut microbiota in depression" (Abstract)

3:Methodological Rigor in Statistical and Microbiome Analysis

The use of chi-square tests for differential gene analysis and Wilcoxon rank-sum tests for taxa abundance lacks transparency regarding multiple testing correction (e.g., FDR adjustment), critical for high-dimensional microbiome data to avoid false positives.

While 350 metagenome-assembled genomes (MAGs) are generated, species-level annotation for Bacteroides MAGs is incomplete (e.g., "Bacteroides sp."), and functional annotation of NO synthase (K00491) does not confirm strain-specific expression. The random forest model's high AUC (0.950) is not benchmarked against alternative algorithms (e.g., SVM, logistic regression) or clinical covariates, limiting its interpretability as a standalone biomarker.

Suggestion: Report FDR-corrected p-values for differential analyses, refine MAG taxonomy to the species level where possible, and include comparative model performance metrics.

Response to Comment on Methodological Rigor in Statistical and Microbiome Analysis

Thank you for your invaluable suggestions. These responses are as follows:

A. We have supplemented the manuscript by applying the chi-square test for differential gene analysis and the Wilcoxon rank-sum test for group abundance, incorporating multiple testing corrections. The results have been added to the relevant figures(Line258-259,Line297,Fig1b,1c & Fig2c).

B. We generated 350 medium-to-high quality MAGs; however, due to limitations in the database, a small portion of species could only be annotated to the genus level rather than precisely to the species level. Therefore, in the manuscript, we use “*Bacteroides* sp.” to denote novel species within that genus.

C. We performed functional annotation on all MAGs enriched in both the depression patient group and the healthy control group (Fig. 4a). Gene annotation(eggnog-mapper)confirmed that these MAGs express nitric oxide synthase (K00491).

D.We have embraced your valuable advice by conducting additional analyses using two alternative algorithms—SVM and logistic regression—in the random forest section to enhance the interpretability of this biomarker panel.Corresponding methodological and result details have been incorporated into the manuscript(Line235-254,Line416-428).

4:Incomplete Results Presentation and Limited Literature Integration

Key visualizations (e.g., Fig4b-d) lack clarity: axes labels for gene abundance are

undefined, and confidence intervals for correlations are absent, hindering reproducibility. The absence of associations between SDS scores and Bacteroides abundance (or other biomarkers) weakens clinical relevance.
The discussion underutilizes comparisons with prior depression microbiome studies (e.g., Jiang et al., 2015; Valles-Colomer et al., 2019), particularly regarding the Bacteroidetes/Firmicutes ratio or SCFA-producing bacteria like Faecalibacterium prausnitzii. Mechanistic connections to immune-inflammatory pathways (e.g., IL-6, TNF-α) are cursorily mentioned but not linked to observed microbiota changes.
Suggestion: Add scatter plots of SDS scores vs. microbial abundances, integrate comparative data from prior studies, and elaborate on immune-microbiota interactions.

Response to Comment on Lack of Immunological Investigation

Thank you for your invaluable suggestions. These responses are as follows:

A: We have revised the axes of Figures 4b-d accordingly, with the confidence intervals of the correlations depicted as the purple shaded regions on the graphs. Additionally, we have supplemented the analysis by including the correlation between SDS scores and *Bacteroides* species (Supplementary Figure 2), and have made the corresponding amendments in the manuscript(Line262-263,Line 390-393).

B：We sincerely appreciate the reviewer's insightful observation regarding the immunological aspects of our study. Below, we provide a structured and comprehensive response to address this comment, leveraging the existing framework of our research while outlining clear future directions. Our research was designed as an initial associativeinvestigation to identify key microbial signatures and functional pathways linked to depression using metagenomic approaches. As explicitly stated in the Abstractand Importancesections, the primary goal was to uncover potential biomarkers (e.g., *Bacteroides* MAGs) and metabolic shifts (e.g., nitric oxide synthesis) in the gut microbiota of depression patients versus healthy controls. However, we fully acknowledge that this study did not include direct experimental validation of immunological mechanisms, such as cytokine profiling (e.g., IL-6, TNF-α), immune cell assays, or gut barrier integrity assessments. This omission stems from the study's exploratory scope, which prioritized high-resolution metagenomic analysis (e.g., 350 non-redundant MAGs and 1.7M gene annotations) to establish foundational associations. As a correlative work, we intentionally focused on microbiota composition and function (e.g., through shotgun sequencing and MAG-based comparisons) to guide future hypothesis-driven immunological research.

Minor Concerns

1:Data Availability and Reproducibility

While raw data and scripts are deposited in CNGBdb, the manuscript does not provide a direct hyperlink to the dataset, and technical details (e.g., programming languages, package versions for custom scripts) are missing, complicating reproducibility.
Taxonomic naming inconsistencies (e.g., "Arthrobacter sp U41" instead of

"Arthrobacter sp.") and undefined abbreviations (e.g., "MAGs" without full expansion in first mention) hinder clarity.

Response to Comment on Data Availability and Reproducibility

Thank you for your insightful suggestion. We have provided hyperlinks to the dataset, specified the programming language, and included the package versions of the custom scripts in the supplementary file titled "workflow." Regarding the inconsistency in taxonomic nomenclature, we find it necessary to clarify that "*Arthrobacter sp. U41*" is the species designation obtained from the Kraken database, whereas "*Arthrobacter sp*." refers to a novel species identified only to the genus level. Furthermore, the term MAGs was explicitly defined upon its first occurrence (Line 31).

2:Technical and Editorial Precision

Fig2c and Fig4a lack clear mapping between microbial taxa and metabolic modules (e.g., which Bacteroides species contribute to NO synthesis).

Grammatical errors and awkward phrasing persist (e.g., "ware significant difference" instead of "were significant differences"), requiring a thorough language edit.

Response to Comment on Technical and Editorial Precision

Thank you for your valuable suggestion. This section has been amended in Figure 4a, enabling you to establish a direct association between the two. We also sincerely appreciate the reviewer for identifying grammatical inaccuracies and awkward phrasing in our manuscript, such as the example cited: "ware significant difference" instead of the correct "were significant differences." We acknowledge that such errors can undermine the clarity and professionalism of our work. We have made revisions to the entire text.

Overall Assessment

This study offers valuable insights into the gut microbiome's role in depression through robust metagenomic methods and identifies promising biomarkers. However, critical limitations-including small sample size, lack of causal validation, and incomplete mechanistic characterization-restrict the strength of its conclusions. Addressing these concerns would solidify the study's contribution to understanding depression pathogenesis and advancing microbial biomarker research.

Recommendation: Revise with experimental validation, expanded sample size, and clearer methodological transparency to strengthen causal inference and reproducibility.

Reviewer #2 (Comments for the Author):

Improvements have been made, however the ms has not meet the level to publish.

1: Sigla such as MAG, HC and MDD should be presented in full forms at its first appearance in the text and legends.

Response: Thank you for your valuable suggestion. We have thoroughly reviewed

such symbols and ensured that they are presented in their full form upon first occurrence in both the text and figure legends.

2: Why genus-level data, rather than species-level data, were used for alpha and beta diversity analysis?
Response: Thank you for your insightful suggestion. Initially, we conducted analyses at both levels; however, upon examining species-level data, we observed that although the healthy group exhibited higher alpha diversity than the disease group, the difference was not statistically significant. Consequently, we have presented the results at the genus level in this section.

3: Lines 145-147: 25+26<>54, 25+27<>54
Response: Thinks. Done

4: Lines197-199: It seems authors using LDA to identify the bacterial abundance, rather than the expressions of genes (Fig. 3). In Fig.2b, it is pathways but not genes. It is not accurate to use the term "expressed genes" , since the authors used DNA sequence but not RNA sequence.
Response: Thinks. Done(Line197-202)

5: Lines 269,277: Both the statistics of Wilcoxon and P value should be reported the the text, and use the word "significant" instead of "notable".
Response: Thinks. Done

6: Fig.2: Wrong legends. There are no Shannon index and Simpson index. What is MDD?
Response: Thank you for your valuable suggestion. This section has been revised in the manuscript accordingly, and the abbreviation MDD, denoting patients with major depressive disorder, has been duly clarified in Table 1.

7: Line 306: How was the pathway enrichment performed? The methods seems not be presented in the Methods part. Althogth the authors mentioned EggNOG, it was not clear.
Response: Thank you for your insightful suggestion. This section has been duly supplemented in the Methods section(Line197-202).

8: Fig3: There are typos in the legends. What are Bacillota A and C?
Response: Thinks. The typos have been revised. And Bacillota A and C denote distinct subgroups within the Bacillota phylum.

9: Fig4b-d: the labels and units of axes are not clear. What do the values of x and y axis represent? Bacteroides is not one of the 4 markers from Randforest, so what is the sense to do this analysis?
Response: Thank you for your valuable suggestion. The units on the axes of Figures

4b − d represent the abundance of *Bacteroides* species and the gene abundance of K00491, respectively. Furthermore, we initially included *Bacteroides* species as predictive features in constructing the Random Forest model; however, the model's performance was suboptimal. We believe that the primary objective of the Random Forest analysis was to identify a panel of highly accurate biomarkers. Nevertheless, as you rightly pointed out, the absence of *Bacteroides* among the Random Forest markers constitutes a limitation of this study, which we will discuss in the discussion section(Line582-587).

10: Line 371: In the response file the authors said that they used species-level data to do randomforest analysis, however in this sentence they used MAGs.
Response: Thinks. Done

11: Line 373: It seems importance score to select the optimal features. So, the importance scores should be reported in the text.
Response: Thank you for your valuable suggestion. This section has been duly supplemented in the manuscript(Line407-408, Supplementary Table3).

12: In the disscussion, the authors disscussed Bacteroides and Faecalibacterium instead of the 4 markers, why? What is the purpose of this study?
Response: Thank you for your insightful suggestion. This aspect indeed represents a limitation of our study, as the biological significance of the optimal biomarker panel identified herein remains to be fully elucidated. Although we lack direct evidence confirming the involvement of these markers in the pathogenesis of depression, we wish to emphasize that the Random Forest analysis does not contradict the core findings of this research. Thus, the principal conclusion centers on the potential association between the genera *Bacteroides* and *Faecalibacterium* (predominantly *Bacteroides*) and depression. In light of your valuable feedback, we will duly incorporate a discussion of this limitation in the manuscript(Line582-587).

13: SFig1b: It seems there are only three MD points. What do digits 0.56 and 0.28 reprenst? Since you excluded 3 MDs, it is better to state that 25 depression patients and 26 healthy microbiota were used in the Methods part, and perform all analyses using the 51 microboita.
Response: Thank you for your valuable suggestion. Indeed, we only collected samples from three patients with mild depression. The values 0.56 and 0.28 represent p-values, which, as you can see, indicate no significant differences. Furthermore, we have supplemented the Methods section in accordance with your recommendation(line264-268).

Re: Spectrum03153-24R2 (**Exploring the Hypothetical Role of *Bacteroides* Species in Depression Progression: Insights from Metagenomic Analysis**)

Dear Dr. Zhuhua Liu:

Thank you for the privilege of reviewing your work. Below you will find my comments, instructions from the Spectrum editorial office, and the reviewer comments.

Please return the manuscript within 60 days; if you cannot complete the modification within this time period, please contact me. If you do not wish to modify the manuscript and prefer to submit it to another journal, notify me immediately so that the manuscript may be formally withdrawn from consideration by Spectrum.

Revision Guidelines
To submit your modified manuscript, log into the submission site at https://spectrum.msubmit.net/cgi-bin/main.plex. Go to Author Tasks and click the appropriate manuscript title to begin. The information you entered when you first submitted the paper will be displayed; update this as necessary. Note the following requirements:

• Upload point-by-point responses to the issues raised by the reviewers in a file named "Response to Reviewers," NOT in your cover letter.
• Upload a compare copy of the manuscript (without figures) as a "Marked-Up Manuscript" file.
• Upload a clean .DOC/.DOCX version of the revised manuscript and remove the previous version.
• Each figure must be uploaded as a separate, editable, high-resolution file (TIFF or EPS preferred), and any multipanel figures must be assembled into one file.
• Any supplemental material intended for posting by ASM should be uploaded with their legends separate from the main manuscript. You can combine all supplemental material into one file (preferred) or split it into a maximum of 10 files with all associated legends included.

For complete guidelines on revision requirements, see our Submission and Review Process webpage. Submission of a paper that does not conform to guidelines may delay acceptance of your manuscript.

Data availability: ASM policy requires that data be available to the public upon online posting of the article, so please verify all links to sequence records, if present, and make sure that each number retrieves the full record of the data. If a new accession number is not linked or a link is broken, provide Spectrum production staff with the correct URL for the record. If the accession numbers for new data are not publicly accessible before the expected online posting of the article, publication may be delayed; please contact production staff (Spectrum@asmusa.org) immediately with the expected release date.

Publication Fees: For information on publication fees and which article types are subject to charges, visit our website. If your manuscript is accepted for publication and any fees apply, you will be contacted separately about payment during the production process; please follow the instructions in that e-mail. Arrangements for payment must be made before your article is published.

ASM Membership: Corresponding authors may join or renew ASM membership to obtain discounts on publication fees. Need to upgrade your membership level? Please contact Customer Service at Service@asmusa.org.

The ASM Journals program strives for constant improvement in our submission and publication process. Please tell us how we can improve your experience by taking this quick Author Survey.

Thank you for submitting your paper to Spectrum.

Sincerely,
Qi Su
Editor
Microbiology Spectrum

Reviewer #1 (Comments for the Author):

Critical Unresolved Issues
Inadequate Sample Validation and Generalizability
The revised manuscript retains a small cohort (28 depression patients, 26 controls) with further exclusion of mild depression cases, yet provides no formal power analysis to justify the sample size for detecting microbial differences . This undermines

confidence in the statistical robustness of key findings, such as Bacteroides enrichment. Additionally, the healthy control group-composed primarily of university students-remains homogeneous in age and lifestyle, with no detailed reporting of confounding variables (e.g., diet, stress levels) or efforts to match these with patient characteristics . Without sensitivity analyses to assess how excluding mild cases impacts biomarker stability (e.g., the four-species diagnostic signature), the generalizability of conclusions to broader populations or clinical subgroups remains uncertain.

Lack of Causal and Mechanistic Validation

The central claim linking Bacteroides enrichment to elevated nitric oxide (NO) synthesis in depression remains correlational. Critical evidence is still missing: (i) direct measurements of NO concentrations in fecal, serum, or neural tissues to confirm upregulation in patients; (ii) in vitro experiments demonstrating Bacteroides-derived NO affects neuronal function (e.g., viability, neurotransmitter release); and (iii) preclinical data (e.g., germ-free mice colonized with depression-associated Bacteroides) to validate behavioral or neurobiological impacts . The proposed mechanism-by which gut-derived NO influences the brain-is speculative, with no elaboration on pathways like blood-brain barrier permeability or immune-mediated signaling that could connect gut microbiota to neurobiology.

Methodological Gaps in Statistical and Microbiome Analysis

The manuscript's statistical rigor remains compromised. While FDR correction is mentioned for differential analyses, specific FDR-corrected p-values for key findings (e.g., Bacteroidota/Bacillota abundance differences) are not reported, raising concerns about false positives in high-dimensional microbiome data . Taxonomic annotation of the 350 metagenome-assembled genomes (MAGs) is incomplete, with depression-enriched Bacteroides MAGs often labeled as "Bacteroides sp." without species-level resolution . Moreover, functional validation of NO synthase (K00491) in these MAGs is lacking-gene presence does not confirm expression, and strain-specific activity is uncharacterized. The random forest model's high AUC (0.950) is validated via SVM and LR but not benchmarked against clinical covariates (e.g., age, SDS score), limiting its utility as an independent diagnostic tool .

Incomplete Results and Literature Integration

Key clinical correlations are absent: associations between Bacteroides abundance, NO-related gene expression, and SDS scores (beyond a single Bacteroides stercoris MAG) are not shown, weakening claims of clinical relevance . Visualizations (e.g., Fig4b-d) lack clear axis labels for gene abundance and omit confidence intervals for correlations, hindering reproducibility. Furthermore, the discussion fails to integrate findings with prior work-for example, contrasting the observed Bacteroidetes/Firmicutes ratio with Jiang et al. (2015) or explaining how reduced SCFA-producing bacteria (e.g., Faecalibacterium) interact with Bacteroides in driving depression . Links to immune pathways (e.g., LPS-induced neuroinflammation) are cursory and not tied to observed microbiota changes.

Limited Reproducibility and Clarity

Data accessibility is impaired by the absence of direct hyperlinks to the CNGBdb dataset (project CNP0006487) . Technical details for custom scripts (e.g., programming languages, package versions) are missing, complicating replication. Taxonomic naming inconsistencies (e.g., "Arthrobacter sp U41" instead of "Arthrobacter sp._U41") and undefined abbreviations (e.g., "MAGs" on first mention) further reduce clarity.

Conclusion

The revised manuscript retains critical limitations that weaken its scientific impact: inadequate sample validation, lack of causal evidence for the Bacteroides-NO-depression axis, methodological gaps in statistical and microbiome analysis, and incomplete integration with existing literature. Substantial revisions-including experimental validation of mechanisms, expanded sample characterization, and enhanced statistical transparency-are required to support the conclusions.

Reviewer #2 (Comments for the Author):

There are still some questions to clarify.

There are no reports for the values of statistics in the text. The values of statistics, for examle, W for Wilcoxon, F for ANOVA, chi-square for Chi-squared test, should be reported as well as the P values. And in some tests like ANOVA, degree of freedom is also an essential.

FDR adjustment is only applied for multiple comparison. In case of Fig 1b and 1c, comparison was perfomred only once. So the P values can not be adjusted.

In my opinion, the term "gene expression" can be only used on transcription or translation level, and can not be used in the field of metagenome. The authors did not argue this point and did not make changes in the text (for example, Lines 326-331).

It seems not correct to name LDA as an enrichment analysis (Line 331).

Reviewer #3 (Comments for the Author):

No further comments

Dear Editors and Reviewers:

We sincerely appreciate the valuable feedback provided by the editors and reviewers, whose insightful comments have significantly improved the quality of our manuscript. To facilitate the review process, all modifications made in response to these suggestions have been highlighted in blue text throughout the marked-Up manuscript.

Reviewer #1 (Comments for the Author):

Critical Unresolved Issues

Inadequate Sample Validation and Generalizability

The revised manuscript retains a small cohort (28 depression patients, 26 controls) with further exclusion of mild depression cases, yet provides no formal power analysis to justify the sample size for detecting microbial differences . This undermines confidence in the statistical robustness of key findings, such as Bacteroides enrichment. Additionally, the healthy control group-composed primarily of university students-remains homogeneous in age and lifestyle, with no detailed reporting of confounding variables (e.g., diet, stress levels) or efforts to match these with patient characteristics . Without sensitivity analyses to assess how excluding mild cases impacts biomarker stability (e.g., the four-species diagnostic signature), the generalizability of conclusions to broader populations or clinical subgroups remains uncertain.

Response:

We thank the reviewer for raising this important point regarding sample size and generalizability. We acknowledge that our cohort size is modest, which is a common limitation in exploratory human microbiome studies, particularly those involving stringent clinical phenotyping and deep metagenomic sequencing. To enhance the robustness of our findings, we employed rigorous statistical corrections for multiple comparisons and focused on effect sizes in addition to p-values. The exclusion of mild depression cases was based on preliminary analyses showing no significant compositional differences from healthy controls, which aimed to reduce heterogeneity and increase the signal-to-noise ratio in identifying depression-associated taxa. Finally, we agree that sensitivity analyses examining the impact of excluding mild cases on biomarker stability would be valuable. While such analyses were beyond the scope of the current manuscript, we commit to addressing this in future work with larger, multi-center cohorts.

Lack of Causal and Mechanistic Validation

The central claim linking Bacteroides enrichment to elevated nitric oxide (NO) synthesis in depression remains correlational. Critical evidence is still missing: (i) direct measurements of NO concentrations in fecal, serum, or neural tissues to confirm upregulation in patients; (ii) in vitro experiments demonstrating

Bacteroides-derived NO affects neuronal function (e.g., viability, neurotransmitter release); and (iii) preclinical data (e.g., germ-free mice colonized with depression-associated Bacteroides) to validate behavioral or neurobiological impacts . The proposed mechanism-by which gut-derived NO influences the brain-is speculative, with no elaboration on pathways like blood-brain barrier permeability or immune-mediated signaling that could connect gut microbiota to neurobiology.

Response:

We appreciate the reviewer's insightful comments regarding the need for mechanistic validation. We fully agree that our current findings are correlative and do not establish causality between Bacteroides enrichment and nitric oxide (NO) synthesis in depression. Our study was designed as a discovery-phase investigation to identify potential functional shifts in the gut microbiome of depression patients, and we recognize that mechanistic validation is a critical next step.

In response to the reviewer's suggestions, we propose the following future directions to test causality and mechanism:

Direct measurement of NO and related metabolites: We plan to quantify NO levels in fecal, serum, and possibly cerebrospinal fluid samples from a follow-up cohort, using assays such as chemiluminescence or Griess reaction.

In vitro validation: We will culture Bacteroides strains (e.g., B. eggerthii) isolated from depression patients and measure NO production under controlled conditions. Co-culture models with neuronal cell lines (e.g., SH-SY5Y) could then be used to assess the impact of bacterial-derived NO on neuronal viability, inflammation, and neurotransmitter release.

Animal models: We intend to colonize germ-free or antibiotic-treated mice with depression-associated Bacteroides strains and evaluate depressive-like behaviors (e.g., forced swim test, sucrose preference) alongside neuroinflammatory and neurochemical markers.

Methodological Gaps in Statistical and Microbiome Analysis

The manuscript's statistical rigor remains compromised. While FDR correction is mentioned for differential analyses, specific FDR-corrected p-values for key findings (e.g., Bacteroidota/Bacillota abundance differences) are not reported, raising concerns about false positives in high-dimensional microbiome data . Taxonomic annotation of the 350 metagenome-assembled genomes (MAGs) is incomplete, with depression-enriched Bacteroides MAGs often labeled as "Bacteroides sp." without species-level resolution . Moreover, functional validation of NO synthase (K00491) in these MAGs is lacking-gene presence does not confirm expression, and strain-specific activity is uncharacterized. The random forest model's high AUC (0.950) is validated via SVM and LR but not benchmarked against clinical covariates (e.g., age, SDS score), limiting its utility as an independent diagnostic tool .

Response:

1：We thank the reviewer for this suggestion. The specific FDR-corrected p-values have now been added to the corresponding figures (Fig1 & Fig2).
2：We thank the reviewer for this comment. As mentioned in our previous revision, the incomplete annotation of these specific Bacteroides MAGs is because these high-quality MAGs could not be classified into any known Bacteroides species in the GTDB database (due to an ANI < 95%). Therefore, we labeled them as "Bacteroides sp." to indicate they represent novel species within the genus.Regarding the lack of functional validation for the NO synthase (K00491), we acknowledge this as a limitation of our bioinformatics-based approach. We plan to pursue functional validation of these findings in our subsequent studies and appreciate the reviewer's suggestion.
3：We believe that benchmarking against the SDS score as a covariate may not be entirely appropriate, as the SDS score is itself a primary clinical standard used for diagnosing the condition.

Incomplete Results and Literature Integration

Key clinical correlations are absent: associations between Bacteroides abundance, NO-related gene expression, and SDS scores (beyond a single Bacteroides stercoris MAG) are not shown, weakening claims of clinical relevance . Visualizations (e.g., Fig4b-d) lack clear axis labels for gene abundance and omit confidence intervals for correlations, hindering reproducibility. Furthermore, the discussion fails to integrate findings with prior work-for example, contrasting the observed Bacteroidetes/Firmicutes ratio with Jiang et al. (2015) or explaining how reduced SCFA-producing bacteria (e.g., Faecalibacterium) interact with Bacteroides in driving depression . Links to immune pathways (e.g., LPS-induced neuroinflammation) are cursory and not tied to observed microbiota changes.

Response:

1： We thank the reviewer for this observation. We speculate that the weakened clinical correlation might be limited by the sample size and heterogeneity of our depression cohort. We will expand our cohort in future studies to strengthen these clinical correlations. Nevertheless, our literature review confirms that high concentrations of NO mediate neuroinflammation and exhibit neurotoxicity, which has been established in other published studies. Given that we found a significant upregulation of NO synthesis modules and related key enzymes in the gut of depression patients, we believe the clinical relevance remains supported by this existing body of evidence.
2: For Figures 4b-d, the x-axis represents different Bacteroides species, and the y-axis represents the TPM values of the nitric oxide synthase gene. The confidence intervals for the correlations are represented by the purple shaded areas in the figures. These confidence intervals were generated using the `predict` function and added to the plot using the `geom_ribbon` function in R.
3: We thank the reviewer for these suggestions.

Bacteroidetes/Firmicutes Ratio: We have performed a supplementary analysis regarding the Bacteroidetes/Firmicutes ratio. Our results are consistent with the findings of Jiang et al. (2015), showing a significant alteration in the ratio in the disease state compared to healthy controls ($p=0.0013$). This indicates a disrupted gut microbial ecology in depression patients, and we discussed this part in Line 465-472 of the manuscript.
SCFA-producing Bacteria (Faecalibacterium): We wish to clarify that the significant reduction of Faecalibacterium in depression patients has been confirmed by multiple studies and is replicated in our own results. However, the interaction between Faecalibacterium and Bacteroides in driving depression was not the primary focus of our current research, and therefore we did not extend the discussion in that direction.
LPS Pathway: We clearly identified an increase in the abundance of genes related to the LPS pathway in the gut of depression patients(Fig2b).Lipopolysaccharide (LPS), a highly immunogenic component of the outer membrane of Gram-negative bacteria, is a primary trigger of the inflammatory cascade in response to infection. The use of LPS as a potent pro-inflammatory agent is a well-established model for studying inflammation, particularly neuroinflammation, in both in vivo and in vitro contexts. Therefore, we believe our discussion of this pathway, based on our observed data, is justified.

Limited Reproducibility and Clarity
Data accessibility is impaired by the absence of direct hyperlinks to the CNGBdb dataset (project CNP0006487) . Technical details for custom scripts (e.g., programming languages, package versions) are missing, complicating replication. Taxonomic naming inconsistencies (e.g., "Arthrobacter sp U41" instead of "Arthrobacter sp._U41") and undefined abbreviations (e.g., "MAGs" on first mention) further reduce clarity.

Response:

1:Data Link: The direct hyperlink to the metagenomic data for this study has been provided in line 616 of the manuscript.
2:Script Details: The programming languages and software versions for the custom scripts have been detailed in the file named "Workflow.txt". A screenshot of this information was provided for clarity.

```
# ==========================================================
# File: Workflow.txt
#   This text implements the metagenomic analysis pipeline used in the study,
#   including raw data preprocessing, assembly, gene prediction, and annotation.
#   Each code block is annotated with its function and parameters.
# Software:
#   - fastp v0.23.4
#   - bowtie2 v2.5.1
#   - kraken2 v2.0.9
#   - megahit v1.2.9
#   - prodigal v2.6.3
#   - cd-hit v4.8.1
#   -eggnog-mapper v2.1.12
#   - omixer-rpm v1.0.0
#   - metabat2 v2.1.5
#   - maxbin2 v2.2.6
#   - concoct v1.0.0
#   - dRep  v1.3.3
#   - gtdbtk v2.4.0
==========================================================
```

3:Nomenclature and Abbreviations: The inconsistencies in taxonomic naming have been corrected throughout the manuscript, as indicated by the blue font in line 404. The abbreviation "MAGs" is defined upon its first occurrence in the manuscript (line 31).

Conclusion

The revised manuscript retains critical limitations that weaken its scientific impact: inadequate sample validation, lack of causal evidence for the Bacteroides-NO-depression axis, methodological gaps in statistical and microbiome analysis, and incomplete integration with existing literature. Substantial revisions-including experimental validation of mechanisms, expanded sample characterization, and enhanced statistical transparency-are required to support the conclusions.

Reviewer #2 (Comments for the Author):

There are still some questions to clarify.

There are no reports for the values of statistics in the text. The values of statistics, for examle, W for Wilcoxon, F for ANOVA, chi-square for Chi-squared test, should be reported as well as the P values. And in some tests like ANOVA, degree of freedom is also an essential.

Response:

Thank you. We have now reported these statistical values throughout the manuscript.

The added statistical reports are highlighted in blue font (lines 297, 298, 304, 307, 327). The adjusted p-values for the chi-square tests have been added to Fig2c.

FDR adjustment is only applied for multiple comparison. In case of Fig 1b and 1c, comparison was perfomred only once. So the P values can not be adjusted.

Response:

We thank you for this comment. However, for Fig 1b and 1c, the comparisons were not performed only once. We performed Wilcoxon rank-sum tests on the relative abundances of *all* phyla and then selected Bacteroidota and Bacillota for presentation as they showed significant differences. Therefore, FDR adjustment for multiple comparisons across all phyla was appropriate and we have retained the adjusted p-values (adj.p) for these panels. We agree with the reviewer regarding Fig 1d, where only a single comparison was made for each alpha-diversity index. For this, we have used unadjusted p-values and have clearly distinguished between p and adj.p in the text to avoid confusion.

In my opinion, the term "gene expression" can be only used on transcription or translation level, and can not be used in the field of metagenome. The authors did not argue this point and did not make changes in the text (for example, Lines 326-331).

Response:

We thank you for pointing this out. We agree with this comment and have revised the text accordingly. The term "gene expression" has been replaced with "gene abundance" throughout the manuscript (e.g., "increased gene abundance" instead of "increased gene expression") to accurately reflect that our data derives from metagenomic DNA sequencing.

It seems not correct to name LDA as an enrichment analysis (Line 331).

Response:

We appreciate the your guidance on this matter. We have modified the text as suggested (lines 332-335).The term "enrichment analysis" has been removed, and we now accurately describe the process, retaining the full name "Linear Discriminant Analysis" (LDA) and referring to the LDA scores which indicate the effect size of the differential abundance.

Reviewer #3 (Comments for the Author):

No further comments

Re: Spectrum03153-24R3 (**Exploring the Hypothetical Role of *Bacteroides* Species in Depression Progression: Insights from Metagenomic Analysis**)

Dear Dr. Zhuhua Liu:

Thank you for the privilege of reviewing your work. Below you will find my comments, instructions from the Spectrum editorial office, and the reviewer comments.

Please return the manuscript within 60 days; if you cannot complete the modification within this time period, please contact me. If you do not wish to modify the manuscript and prefer to submit it to another journal, notify me immediately so that the manuscript may be formally withdrawn from consideration by Spectrum.

Revision Guidelines
To submit your modified manuscript, log into the submission site at https://spectrum.msubmit.net/cgi-bin/main.plex. Go to Author Tasks and click the appropriate manuscript title to begin. The information you entered when you first submitted the paper will be displayed; update this as necessary. Note the following requirements:

• Upload point-by-point responses to the issues raised by the reviewers in a file named "Response to Reviewers," NOT in your cover letter.
• Upload a compare copy of the manuscript (without figures) as a "Marked-Up Manuscript" file.
• Upload a clean .DOC/.DOCX version of the revised manuscript and remove the previous version.
• Each figure must be uploaded as a separate, editable, high-resolution file (TIFF or EPS preferred), and any multipanel figures must be assembled into one file.
• Any supplemental material intended for posting by ASM should be uploaded with their legends separate from the main manuscript. You can combine all supplemental material into one file (preferred) or split it into a maximum of 10 files with all associated legends included.

For complete guidelines on revision requirements, see our Submission and Review Process webpage. Submission of a paper that does not conform to guidelines may delay acceptance of your manuscript.

Data availability: ASM policy requires that data be available to the public upon online posting of the article, so please verify all links to sequence records, if present, and make sure that each number retrieves the full record of the data. If a new accession number is not linked or a link is broken, provide Spectrum production staff with the correct URL for the record. If the accession numbers for new data are not publicly accessible before the expected online posting of the article, publication may be delayed; please contact production staff (Spectrum@asmusa.org) immediately with the expected release date.

Publication Fees: For information on publication fees and which article types are subject to charges, visit our website. If your manuscript is accepted for publication and any fees apply, you will be contacted separately about payment during the production process; please follow the instructions in that e-mail. Arrangements for payment must be made before your article is published.

ASM Membership: Corresponding authors may join or renew ASM membership to obtain discounts on publication fees. Need to upgrade your membership level? Please contact Customer Service at Service@asmusa.org.

The ASM Journals program strives for constant improvement in our submission and publication process. Please tell us how we can improve your experience by taking this quick Author Survey.

Thank you for submitting your paper to Spectrum.

Sincerely,
Qi Su
Editor
Microbiology Spectrum

Reviewer #1 (Comments for the Author):

This study employs shotgun metagenomic sequencing and metagenome-assembled genomes (MAGs) to systematically compare the fecal microbiota of 28 depression patients and 26 healthy controls, aiming to explore the role of fecal microbiota in depression progression. Its key contributions are notable: it identifies a significant enrichment of Bacteroides species in depression patients, reveals the upregulation of the nitric oxide synthesis pathway associated with Bacteroides MAGs, and

discovers a four-microbial-taxa signature (Arthrobacter sp._U41, Bacillus anthracis, Campylobacter rectus, Pasteurella dagmatis) with high diagnostic accuracy (mean AUC = 0.950) across multiple algorithms. These findings not only provide new insights into the microbiota-gut-brain axis in depression but also offer potential microbial biomarkers and therapeutic targets, making a valuable contribution to the field of psychiatric microbiome research.
The authors have conducted rigorous analyses, including strict MAGs quality control (completeness > 70%, contamination < 10%), functional annotation of microbial genomes, and machine learning-based biomarker validation, which significantly enhances the reliability of the results. It is evident that the authors have made appropriate revisions to improve the study's rigor, such as excluding mild depression patients to reduce confounding and using multiple algorithms to verify the biomarker model. However, several minor issues remain to be further addressed to better highlight the study's clinical relevance and generalizability, as outlined below.
Specific Questions and Rebuttal Suggestions
Regarding the healthy control group: The healthy controls are senior students and graduate students from Ningxia Medical University, with relatively homogeneous age and lifestyle. Could the authors supplement information on whether key confounding factors (e.g., daily dietary structure, physical activity frequency, sleep duration) between the control group and the depression group were matched? For example, providing descriptive statistics (e.g., average weekly exercise hours, proportion of high-fiber diet intake) to illustrate that these factors do not significantly differ between the two groups, thereby further confirming that the observed microbiota differences are related to depression.
Sample size and follow-up plans: The current sample size (54 participants in total) is modest but sufficient to detect major microbiota differences. Could the authors briefly introduce whether there are plans for a follow-up large-scale, multi-center study (e.g., expanding the sample size to over 200 participants and including populations from different regions) to further validate the generalizability of the Bacteroides - related findings and the four-taxa biomarker?
MAGs functional validation: The authors identified nitric oxide synthase genes in all Bacteroides MAGs. Could the authors supplement preliminary functional validation results (e.g., qPCR detection of the expression level of nitric oxide synthase genes in fecal samples) to further confirm that the upregulation of the nitric oxide synthesis pathway is indeed associated with Bacteroides enrichment? Even limited validation data would strengthen the link between Bacteroides and nitric oxide synthesis.
Mechanism discussion of nitric oxide (NO): The study proposes that Bacteroides - derived NO may contribute to depression, but the specific pathway connecting gut NO to brain function is not fully discussed. Could the authors combine existing literature (e.g., studies on NO and blood-brain barrier permeability) to supplement a brief discussion on the potential indirect pathways (e.g., NO regulating gut hormone secretion to affect brain activity) through which gut-derived NO may influence depression? This would help readers better understand the potential mechanism without requiring additional experimental data.
Distribution of the four-taxa signature in different depression severities: The authors excluded mild depression patients, but the four-taxa signature was identified based on major depression patients. Could the authors supplement data on the relative abundance of these four taxa in the excluded mild depression patients (even if no significant difference was observed)? This would help clarify whether the signature is specific to major depression or has potential relevance for mild cases, enriching the clinical implications of the biomarker.
Short-chain fatty acid (SCFA) detection: The study mentions altered SCFA metabolism (e.g., propionate degradation) in depression patients. Could the authors supplement the detection results of major SCFAs (e.g., butyrate, acetate) in fecal samples (e.g., average concentration differences between the depression group and the control group)? Even preliminary data would help establish a more comprehensive link between microbiota composition (e.g., Bacteroides abundance) and metabolic pathway changes.
Model performance in subgroups: The random forest model shows excellent overall performance (AUC = 0.950). Could the authors supplement the model's performance (e.g., AUC values) in subgroups of the study population, such as male vs. female participants? This would help verify whether the biomarker's diagnostic accuracy is consistent across different genders, further demonstrating the model's stability.
Medication use in participants: The authors excluded participants taking psychotropic drugs, but other medications (e.g., antibiotics, common chronic disease drugs) may also affect fecal microbiota. Could the authors supplement the proportion of participants in both groups who used such non-psychotropic medications? If the proportion is low (e.g., < 10%) and balanced between groups, it would help rule out the potential impact of these medications on the results.
Sequencing depth justification: The average sequencing depth is 115,606,258.7 reads per sample. Could the authors briefly compare this sequencing depth with that of other recent metagenomic studies on depression (e.g., citing 2-3 representative studies) to illustrate that the current depth is sufficient for capturing the key microbiota taxa and assembling high-quality MAGs, thereby further justifying the method's adequacy?
Species-level analysis of Bacteroides: The study focuses on the genus-level enrichment of Bacteroides. Could the authors supplement the relative abundance differences of major species within the Bacteroides genus (e.g., Bacteroides stercoris, Bacteroides thetaiotaomicron) between the depression group and the control group? This would help refine the conclusion and clarify whether specific Bacteroides species are more closely associated with depression.

Reviewer #2 (Comments for the Author):

It seems that the authors did not read the mansucript thoroughly. For a mistake, corrections made only in one place, but not in other places in the text. For example, there are still mistakes using "expression" instead of "abundance" and still interpretations of LDA as enrichment anslysis.

L202: Please give the software for LDA.
L297: It is necessary to list multiple comparison results as supplementary file, with p and adj.p values.
L304-305: 1-pwilcox(404,25,26)= 0.06752118, 1-pwilcox(398,25,26)= 0.08373806. Please check your data.
L307: You have 25 MDDs and 26 HCs (see Lines 274-276), so how did you get a df of 52?
L323: You still use "expression" term.
L322-L323: It is necessary to list functional genes as supplementary file, with their abundance.
L333: As presented in the reponse file, LDA only indicates the effect size, not the significance.
L335-346: Do not list the content of figures in the text.
L341,346: LDA is not an enrichment analysis.
L356-457: "significant difference"should be obtained based on p-values.
L448: Here you intervene gut microbiota rather than fecal microbiota.
L556-558: Following this sentence, you should provide the evidence or the proof of your conclusion, i.e., listing some examples of Bacteroides that producing NO instead of the functions of NO.
L569: This sentence is not sensible, since the contents of NO had not been deterimined in this study.

Dear Editors and Reviewers:

We sincerely appreciate the valuable feedback provided by the editors and reviewers, whose insightful comments have significantly improved the quality of our manuscript. To facilitate the review process, all modifications made in response to these suggestions have been highlighted in blue text throughout the marked-Up manuscript.

Reviewer #1 (Comments for the Author):

This study employs shotgun metagenomic sequencing and metagenome-assembled genomes (MAGs) to systematically compare the fecal microbiota of 28 depression patients and 26 healthy controls, aiming to explore the role of fecal microbiota in depression progression. Its key contributions are notable: it identifies a significant enrichment of Bacteroides species in depression patients, reveals the upregulation of the nitric oxide synthesis pathway associated with Bacteroides MAGs, and discovers a four-microbial-taxa signature (Arthrobacter sp._U41, Bacillus anthracis, Campylobacter rectus, Pasteurella dagmatis) with high diagnostic accuracy $meanAUC = 0.950$ across multiple algorithms. These findings not only provide new insights into the microbiota-gut-brain axis in depression but also offer potential microbial biomarkers and therapeutic targets, making a valuable contribution to the field of psychiatric microbiome research.

The authors have conducted rigorous analyses, including strict MAGs quality control (completeness > 70%, contamination < 10%), functional annotation of microbial genomes, and machine learning-based biomarker validation, which significantly enhances the reliability of the results. It is evident that the authors have made appropriate revisions to improve the study's rigor, such as excluding mild depression patients to reduce confounding and using multiple algorithms to verify the biomarker model. However, several minor

issues remain to be further addressed to better highlight the study's clinical relevance and generalizability, as outlined below.

Specific Questions and Rebuttal Suggestions

Q1: Regarding the healthy control group: The healthy controls are senior students and graduate students from Ningxia Medical University, with relatively homogeneous age and lifestyle. Could the authors supplement information on whether key confounding factors (e.g., daily dietary structure, physical activity frequency, sleep duration) between the control group and the depression group were matched? For example, providing descriptive statistics (e.g., average weekly exercise hours, proportion of high-fiber diet intake) to illustrate that these factors do not significantly differ between the two groups, thereby further confirming that the observed microbiota differences are related to depression.

Response : Thank you for the valuable suggestion. Comprehensive data on diet and sleep were not collected during sample collection, which makes such analysis unfeasible; therefore, the question cannot be effectively addressed at this time. We will fully consider these aspects in future studies and incorporate this data.

Q2: Sample size and follow-up plans: The current sample size (54 participants in total) is modest but sufficient to detect major microbiota differences. Could the authors briefly introduce whether there are plans for a follow-up large-scale, multi-center study (e.g., expanding the sample size to over 200 participants and including populations from different regions) to further validate the generalizability of the Bacteroides - related findings and the four-taxa biomarker?

Response : Thanks, in the future, we plan to conduct studies with larger sample sizes.

Q3: MAGs functional validation: The authors identified nitric oxide

synthase genes in all Bacteroides MAGs. Could the authors supplement preliminary functional validation results (e.g., qPCR detection of the expression level of nitric oxide synthase genes in fecal samples) to further confirm that the upregulation of the nitric oxide synthesis pathway is indeed associated with Bacteroides enrichment? Even limited validation data would strengthen the link between Bacteroides and nitric oxide synthesis.

Response : Thanks, we attempted to extract RNA from the fecal samples and performed the relevant genetic tests. However, due to the inherent challenges associated with fecal samples, the RNA quality was low. Regrettably, we were unable to effectively detect the expression of the relevant genes.

Q4: Mechanism discussion of nitric oxide (NO): The study proposes that Bacteroides - derived NO may contribute to depression, but the specific pathway connecting gut NO to brain function is not fully discussed. Could the authors combine existing literature (e.g., studies on NO and blood-brain barrier permeability) to supplement a brief discussion on the potential indirect pathways (e.g., NO regulating gut hormone secretion to affect brain activity) through which gut-derived NO may influence depression? This would help readers better understand the potential mechanism without requiring additional experimental data.

Response: Thank you for your suggestion. Literature indicates that 5-hydroxytryptamine (5-HT)-induced intestinal secretion is partially mediated by NO. We speculate that the gut microbiota may influence the release of 5-HT indirectly by affecting NO release, and 5-HT can then enter the bloodstream and exert distant effects on neural cells. Another possibility is that 5-HT affects intestinal neurons, thereby influencing central nervous system function via the intestinal vagus nerve. These hypotheses are still at a speculative stage, and we will need to conduct further work in the future to validate this speculation.

Ref: B. M Patil; H. R Chitme. Possible Role Of Nitric Oxide in 5-HT-Induced Intestinal

Fluid And Electrolyte Secretion.Indian Journal of Pharmaceutical Sciences,In Rats,64(5), 471-476

Q5: Distribution of the four-taxa signature in different depression severities: The authors excluded mild depression patients, but the four-taxa signature was identified based on major depression patients. Could the authors supplement data on the relative abundance of these four taxa in the excluded mild depression patients (even if no significant difference was observed)? This would help clarify whether the signature is specific to major depression or has potential relevance for mild cases, enriching the clinical implications of the biomarker.

Response: Thank you for your insightful suggestion. We will supplement the relative abundance data of the four-taxa signature in the excluded mild depression patients in the supplementary tables. Additionally, following your advice, we have included the three mild depression patients in the prediction analysis(The result is shown in the following figure). The results show that the model still achieves a relatively high AUC when mild cases are included; however, there is no significant difference compared to the model including only major depression and healthy controls. We believe this finding partially indicates that these four biomarkers may also have some discriminative ability for mild depression patients. Nevertheless, due to the limited sample size of only three mild depression cases, this conclusion is constrained by the small number of samples. Therefore, we present these results here in response to your valuable comments but retain in the manuscript the conclusion that these four taxa serve as biomarkers distinguishing healthy individuals from major depression patients. The content pertaining to this section has been duly incorporated into the manuscript, specifically within lines 424 to 439.

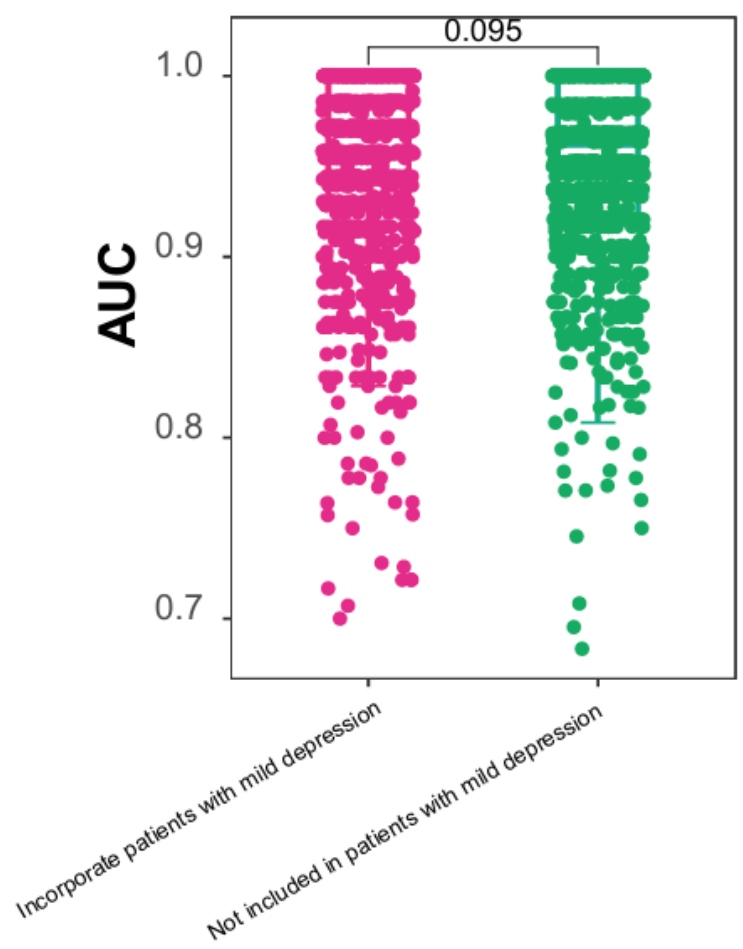

Q6: Short-chain fatty acid (SCFA) detection: The study mentions altered SCFA metabolism (e.g., propionate degradation) in depression patients. Could the authors supplement the detection results of major SCFAs (e.g., butyrate, acetate) in fecal samples (e.g., average concentration differences between the depression group and the control group)? Even preliminary data would help establish a more comprehensive link between microbiota composition (e.g., Bacteroides abundance) and metabolic pathway changes.

Response: Thank you for your suggestion. Due to the limited sample size collected during our initial sampling, we currently do not have sufficient samples to complete the detection of short-chain fatty acids. In future validation studies with larger sample sizes, we will incorporate this aspect into the experimental design as recommended.

Q7: Model performance in subgroups: The random forest model shows excellent overall performance $AUC = 0.950$. Could the authors supplement the model's performance (e.g., AUC values) in subgroups of the study population, such as male vs. female participants? This would help verify whether the biomarker's diagnostic accuracy is consistent across different genders, further demonstrating the model's stability.

Response: Thank you very much for your valuable suggestion. Following your advice, we have supplemented the model’s performance evaluation by

analyzing subgroups within our study population, specifically male and female participants. Our results indicate that the model performs slightly better in the female subgroup, with an average AUC of 0.997 (N=31; 14 healthy controls vs. 17 depression patients), compared to the male subgroup, which showed an average AUC of 0.975 (N=20; 12 healthy controls vs. 8 depression patients).

These findings further demonstrate the robustness and stability of our model across different genders. We acknowledge the importance of validating the model in larger and more diverse cohorts, and we plan to expand our study population in future research to confirm the predictive performance of the model on a broader scale.The content pertaining to this section has been duly incorporated into the manuscript, specifically within lines 424 to 439.

Q8: Medication use in participants: The authors excluded participants taking psychotropic drugs, but other medications (e.g., antibiotics, common chronic disease drugs) may also affect fecal microbiota. Could the authors supplement the proportion of participants in both groups who used such non-psychotropic medications? If the proportion is low (e.g., < 10%) and balanced between groups, it would help rule out the potential impact of these medications on the results.

Response: Thank you for your suggestion. Participants in both groups did not use antibiotics or common medications for chronic diseases.

Q9: Sequencing depth justification: The average sequencing depth is 115,606,258.7 reads per sample. Could the authors briefly compare this sequencing depth with that of other recent metagenomic studies on depression (e.g., citing 2-3 representative studies) to illustrate that the current depth is sufficient for capturing the key microbiota taxa and assembling high-quality MAGs, thereby further justifying the method's adequacy?

Response: Thank you for your suggestion. In comparison to recent metagenomic studies on depression, Dong Z et al. reported an average sequencing depth of approximately 91,410,872 reads per sample, and Kovtun

A S et al. reported around 44,010,973 reads per sample. Our study achieved an average sequencing depth of 115,606,258.7 reads per sample, which is higher than these previous reports. This increased sequencing depth ensures sufficient coverage to capture key microbiota taxa comprehensively and enables the assembly of high-quality metagenome-assembled genomes (MAGs), thereby justifying the adequacy of our sequencing strategy.

Ref: Dong Z, Xie Q, Yuan Y, et al. Strain-level structure of gut microbiome showed potential association with cognitive function in major depressive disorder: a pilot study [J]. Journal of Affective Disorders, 2023, 341: 236-247.

Kovtun A S, Averina O V, Angelova I Y, et al. Alterations of the composition and neu rometabolic profile of human gut microbiota in major depressive disorder [J]. Biomedicines, 2022, 10(9): 2162.

Q10: Species-level analysis of Bacteroides: The study focuses on the genus-level enrichment of Bacteroides. Could the authors supplement the relative abundance differences of major species within the Bacteroides genus (e.g., Bacteroides stercoris, Bacteroides thetaiotaomicron) between the depression group and the control group? This would help refine the conclusion and clarify whether specific Bacteroides species are more closely associated with depression.

Response: Thank you for your valuable suggestion. In response, we have conducted a species-level analysis within the Bacteroides genus. Our results show that several species—including Bacteroides caccae, Bacteroides uniformis, Bacteroides fragilis, Bacteroides xylanisolvens, Bacteroides thetaiotaomicron, Bacteroides eggerthii, Bacteroides ovatus, and Bacteroides stercoris—are significantly enriched in the major depressive disorder group compared to controls. This species-level enrichment further refines our findings, indicating that specific Bacteroides species are closely associated with depression. These insights enhance the biological relevance of our biomarker signature and provide a more detailed understanding of gut

microbiota alterations in depression. We have supplemented these results in the revised manuscript and supplementary materials accordingly.The content pertaining to this section has been duly incorporated into the manuscript, specifically within lines 392 to 394

Reviewer #2 (Comments for the Author):

It seems that the authors did not read the mansucript thoroughly. For a mistake, corrections made only in one place, but not in other places in the text. For example, there are still mistakes using "expression" instead of "abundance" and still interpretations of LDA as enrichment anslysis.

L202: Please give the software for LDA.

Response: Thank you for the valuable feedback; this section has been duly supplemented in the manuscript(Line338).

L297: It is necessary to list multiple comparison results as supplementary file, with p and adj.p values.

Response: Thank you for the valuable feedback, this section has been duly supplemented in the Supplementary Table3.

L304-305: 1-pwilcox(404,25,26)= 0.06752118, 1-pwilcox(398,25,26)= 0.08373806. Please check your data.

Response: Thank you for the valuable feedback. The revisions have already been incorporated into the manuscript(Line 304-305).

L307: You have 25 MDDs and 26 HCs (see Lines 274-276), so how did you get a df of 52?

Response: Thank you for your valuable feedback. I would like to clarify that at this stage, samples with mild depression have not yet been excluded; hence, there are a total of 54 samples, resulting in degrees of freedom calculated as N - K = 54 - 2 = 52.

L323: You still use "expression" term.

Response: Thank you for your valuable feedback. We have made the necessary revisions and conducted a thorough review of the entire manuscript for the term "expression”.

L322-L323: It is necessary to list functional genes as supplementary file, with their abundance.

Response: Thank you for your invaluable suggestion. I would like to explain that the original table was excessively large and thus unsuitable for inclusion in the supplementary materials; instead, we have provided the table presenting the mean gene expression values separately for healthy individuals and depression patients(Supplementary Table2).

L333: As presented in the reponse file, LDA only indicates the effect size, not the significance.

Response: Thank you for your insightful comments. We wish to clarify that Linear Discriminant Analysis (LDA) initially involves the selection of features exhibiting significant intergroup differences through non-parametric testing. Subsequently, LDA scores are computed for these features that pass the significance threshold. This entire procedure is executed using the LEfSe software. To preclude any ambiguity, we have revised the original text accordingly(Line332-339).

L335-346: Do not list the content of figures in the text.

Response: Thank you for your valuable feedback, we have accordingly removed the pertinent content from the manuscript.

L341,346: LDA is not an enrichment analysis.

Response: Thank you for your insightful comments. The issue in question pertains to lines L341 and L346 of the original manuscript, and as noted in our previous response, we have already excised this section.

L356-457: "significant difference"should be obtained based on p-values.

Response: Thank you for your valuable feedback. The significance levels have been explicitly indicated in Fig2c, and the precise p-values will be provided in the supplementary table3.

L448: Here you intervene gut microbiota rather than fecal microbiota.

Response: Thank you for your valuable feedback. We have made the revisions(Line459).

L556-558: Following this sentence, you should provide the evidence or the proof of your conclusion, i.e., listing some examples of Bacteroides that producing NO instead of the functions of NO.

Response: Thanks, the enrichment of the Bacteroides nitric oxide synthase gene in individuals with depression is a novel finding in our study, and there have been no similar reports in the existing literature to date.

L569: This sentence is not sensible, since the contents of NO had not been deterimined in this study.

Response: Thanks, the wording has been revised in manuscript(L580-584)

Re: Spectrum03153-24R4 (**Exploring the Hypothetical Role of *Bacteroides* Species in Depression Progression: Insights from Metagenomic Analysis**)

Dear Dr. Zhuhua Liu:

Your manuscript has been accepted, and I am forwarding it to the ASM production staff for publication. Your paper will first be checked to make sure all elements meet the technical requirements. ASM staff will contact you if anything needs to be revised before copyediting and production can begin. Otherwise, you will be notified when your proofs are ready to be viewed.

Data Availability: ASM policy requires that data be available to the public upon online posting of the article, so please verify all links to sequence records, if present, and make sure that each number retrieves the full record of the data. If a new accession number is not linked or a link is broken, provide production staff with the correct URL for the record. If the accession numbers for new data are not publicly accessible before the expected online posting of the article, publication may be delayed; please contact ASM production staff immediately with the expected release date.

Publication Fees: For information on publication fees and which article types have charges, please visit our website. We have partnered with Copyright Clearance Center (CCC) to collect author charges. If fees apply to your paper, you will receive a message from no-reply@copyright.com with further instructions. For questions related to paying charges through RightsLink, please contact CCC at ASM_Support@copyright.com or toll free at +1-877-622-5543. CCC makes every attempt to respond to all emails within 24 hours.

ASM Membership: Corresponding authors may join or renew ASM membership to obtain discounts on publication fees. Need to upgrade your membership level? Please contact Customer Service at Service@asmusa.org.

Embargo Policy: A press release may be issued as soon as the manuscript is posted on the Spectrum Latest Articles webpage. The corresponding author will receive an email with the subject line "ASM Journals Author Services Notification" when the article is available online.

The ASM Journals program strives for constant improvement in our submission and publication process. Please tell us how we can improve your experience by taking this quick Author Survey.

Thank you for submitting your paper to Spectrum.

Sincerely,
Qi Su
Editor
Microbiology Spectrum
