# Supplementary material for: Exploring the hypothetical role of Bacteroides species in depression progression: insights from metagenomic analysis
Source: Microbiol Spectr. 2026 Aug 11;14(9):e03153-24. doi: 10.1128/spectrum.03153-24 (PMC13532182; doi:10.1128/spectrum.03153-24)
Supplement: Supplementary file 2 [file spectrum.03153-24-s0001.pdf]

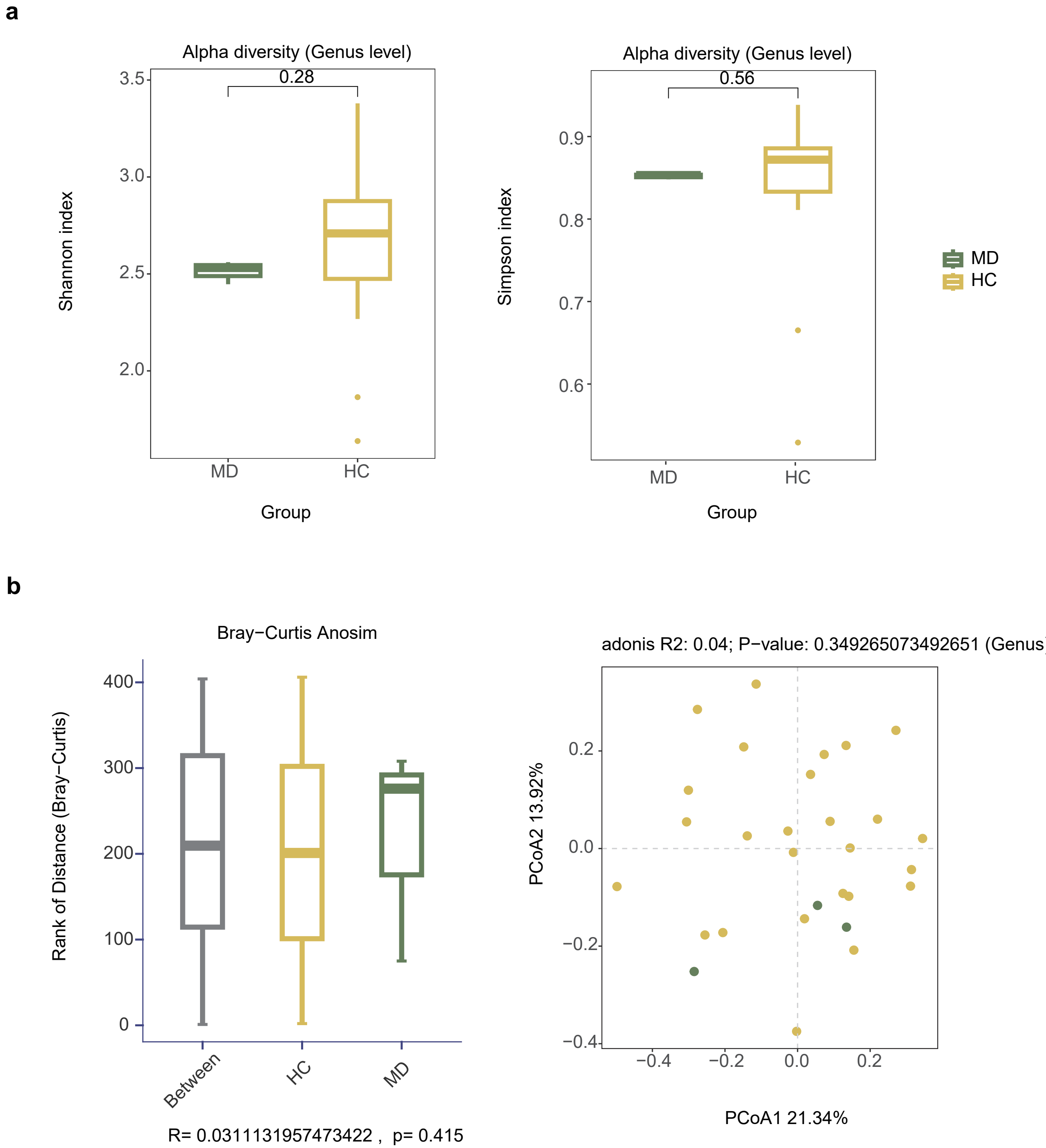

Sfig1 Gut microbiota composition analysis in mild depression people and healthy controls (a)The difference in Shannon index and Simpson index at the genus level in the mild depression and control groups (Wilcoxon test $p < 0.05$).(b)The difference in β diversity by principal coordinate analysis at the genus level in the mild depression and control groups (PERMANOVA,$p < 0.05$). Yellow represents the healthy group, while green represents the mild depression group.
