# Supplementary material for: Exploring the hypothetical role of Bacteroides species in depression progression: insights from metagenomic analysis
Source: Microbiol Spectr. 2026 Aug 11;14(9):e03153-24. doi: 10.1128/spectrum.03153-24 (PMC13532182; doi:10.1128/spectrum.03153-24)
Supplement: Supplementary file 3 [file spectrum.03153-24-s0002.pdf]

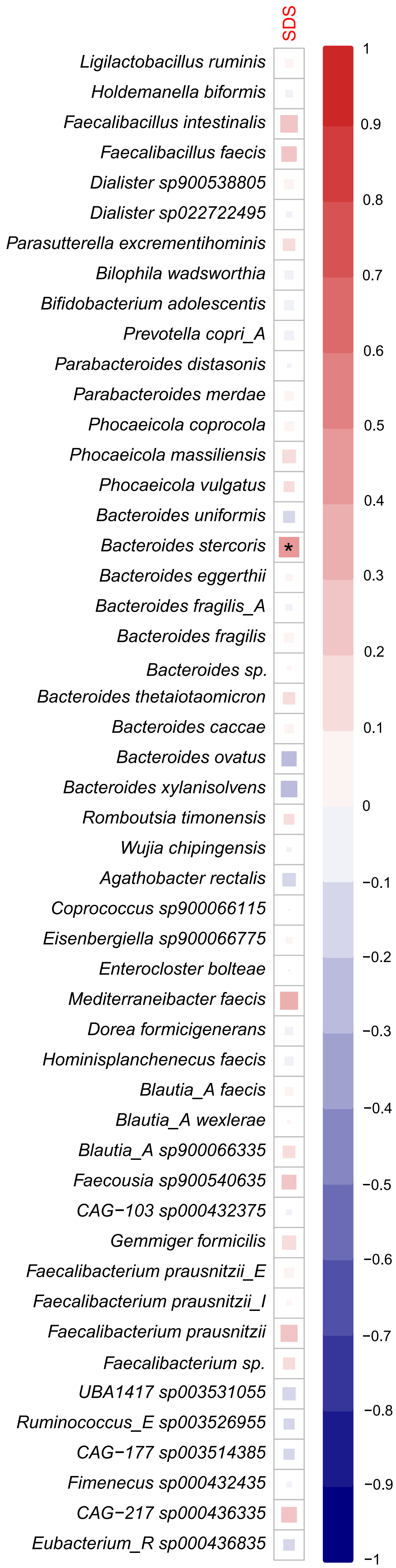

Sfig2 Correlation between differential MAGs and SDS scores in patients with depression (*p<0.05)
