# Supplementary material for: Exploring the hypothetical role of Bacteroides species in depression progression: insights from metagenomic analysis
Source: Microbiol Spectr. 2026 Aug 11;14(9):e03153-24. doi: 10.1128/spectrum.03153-24 (PMC13532182; doi:10.1128/spectrum.03153-24)
Supplement: Supplementary file 4 [file spectrum.03153-24-s0003.pdf]

a

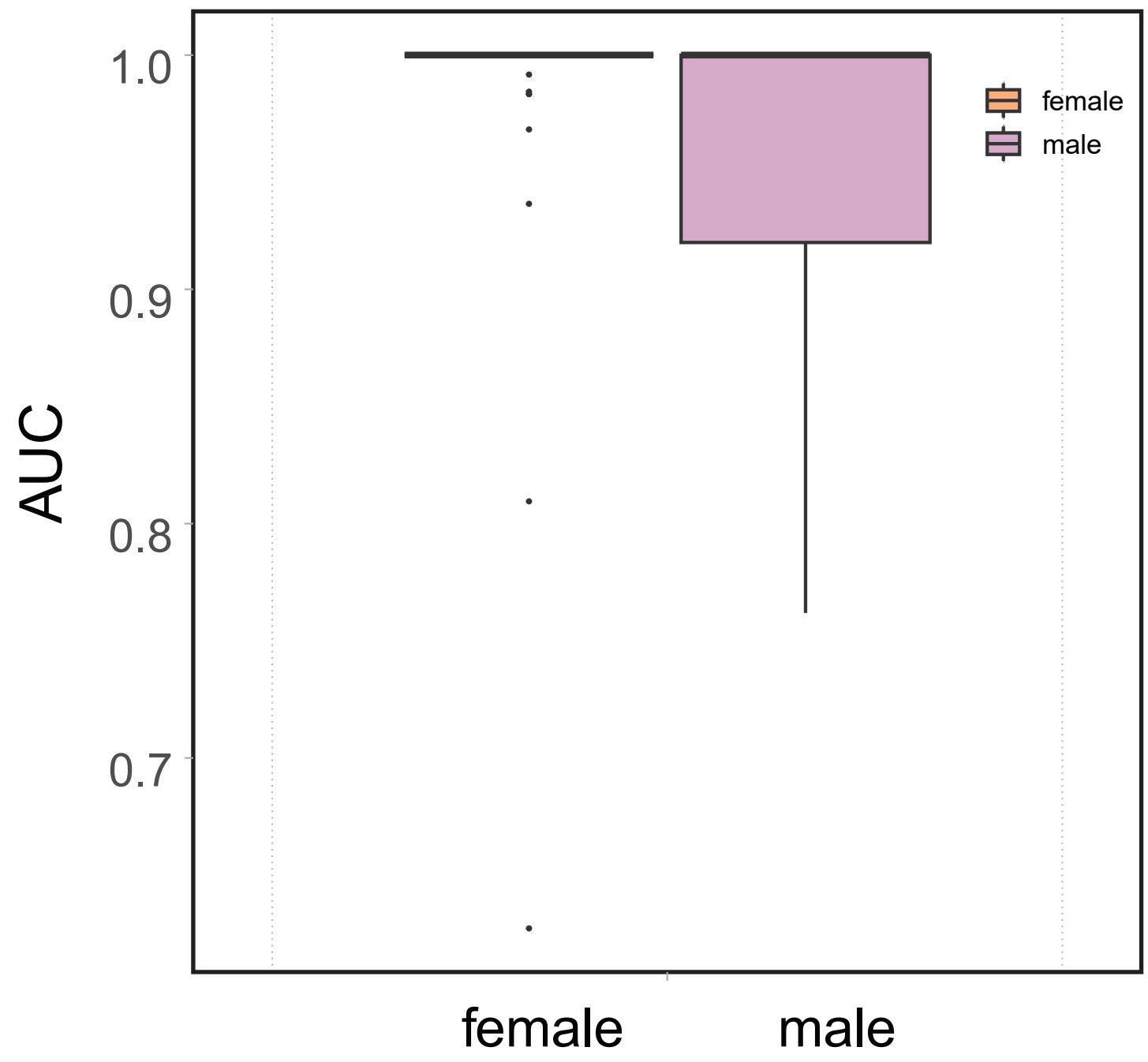

b

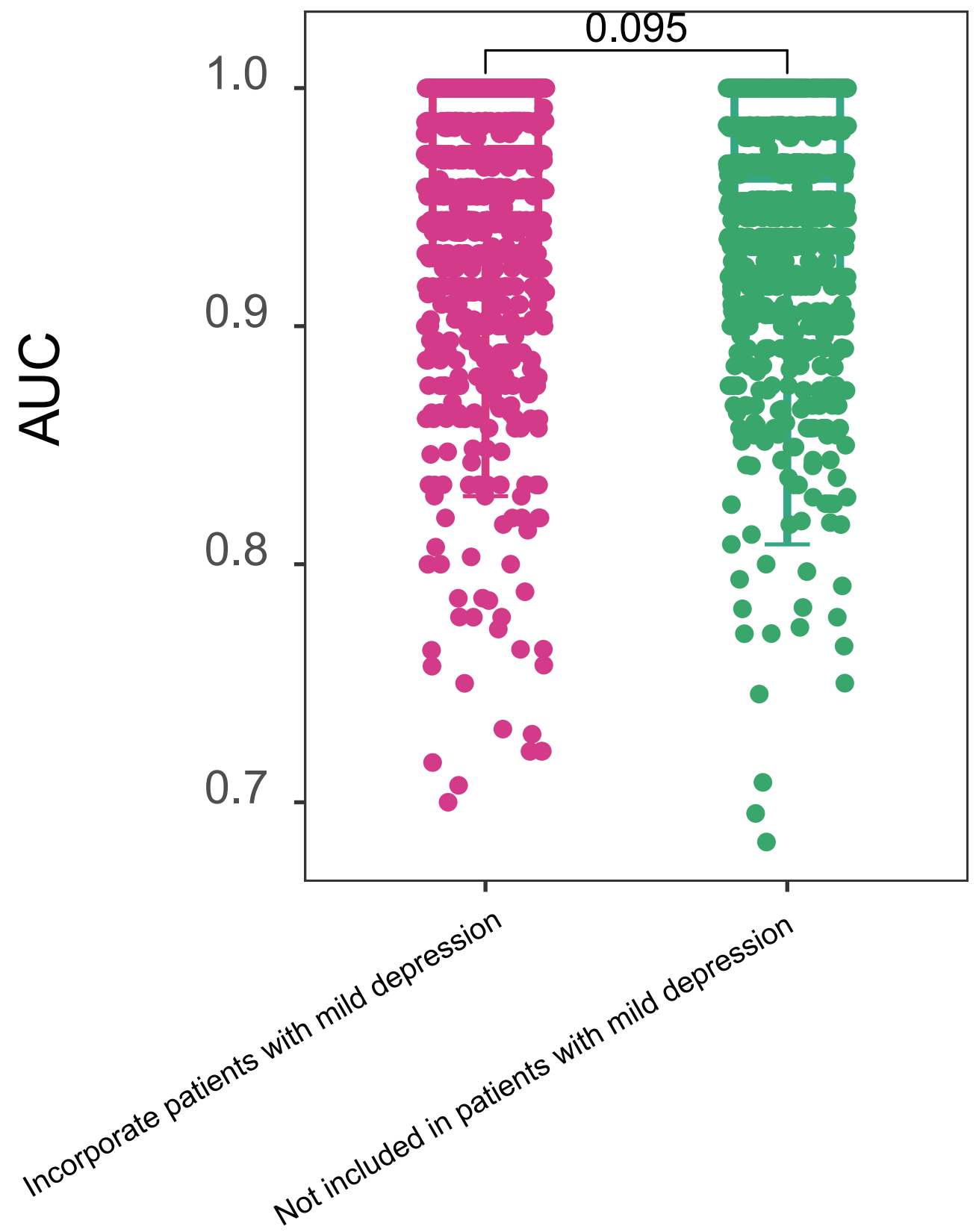

Sfig3

(a) Predictive performance of the random forest model across distinct subgroups (male versus female);
(b) Comparative analysis of the random forest model's predictive efficacy with and without the inclusion of mild depression patients in the cohort (1,000 runs).
